# Therapeutic potential of an intestinotrophic hormone, glucagon-like peptide 2, for treatment of type 2 short bowel syndrome rats with intestinal bacterial and fungal dysbiosis

**DOI:** 10.1186/s12879-021-06270-w

**Published:** 2021-06-16

**Authors:** Xiuting Hu, Wei Cheng, Shengxian Fan, Yuhua Huang, Xi Chen, Zhiwei Jiang, Jian Wang

**Affiliations:** 1 State Key Laboratory of Pharmaceutical Biotechnology, School of Life Sciences, Nanjing University, 163, Xianli Avenue, Nanjing, 210000 China; 2 Department of General Surgery, Jiangsu Province Hospital of Chinese Medicine, Affiliated Hospital of Nanjing University of Chinese Medicine, Nanjing, 210000 China; 3 Department of General Surgery, Nanjing Drum Tower Hospital, Medical School of Nanjing University, Nanjing, 210008 China; 4 Department of General Surgery, Jinling Hospital, Medical School of Nanjing University, Nanjing, 210000 China; 5 Department of Surgery, Fourth Affiliated Hospital of Nanjing Medical University, Nanjing, 210000 China

**Keywords:** Intestinal bacterial and fungal, Dysbiosis, Short bowel syndrome, Intestinotrophic hormone, Glucagon-like peptide 2

## Abstract

**Background:**

Previous studies showed that type 2 short bowel syndrome (SBS) rats were accompanied by severe intestinal bacterial dysbiosis. Limited data are available for intestinal fungal dysbiosis. Moreover, no effective therapeutic drugs are available for these microbiota dysbiosis. The aims of our study were to investigate the therapeutic potential of glucagon-like peptide 2 (GLP-2) for these microbiota dysbiosis in type 2 SBS rats.

**Methods:**

8-week-old male SD rats which underwent 80% small bowel resection, ileocecum resection, partial colon resection and jejunocolostomy, were treated with saline (SBS group, *n* = 5) or GLP-2 (GLP2.SBS group, *n* = 5). The Sham group rats which underwent transection and re-anastomosis were given a saline placebo (Sham group, *n* = 5). 16S rRNA and ITS sequencing were applied to evaluate the colonic bacterial and fungal composition at 22 days after surgery, respectively.

**Results:**

The relative abundance of Actinobacteria, Firmicutes and proinflammatory Proteobacteria increased significantly in SBS group rats, while the relative abundance of Bacteroidetes, Verrucomicrobia and Tenericutes decreased remarkably. GLP-2 treatment significantly decreased Proteus and increased Clostridium relative to the saline treated SBS rats. The diversity of intestinal fungi was significantly increased in SBS rats, accompanied with some fungi abnormally increased and some resident fungi (e.g., Penicillium) significantly decreased. GLP-2 treatment significantly decreased Debaryomyces and Meyerozyma, and increased Penicillium. Moreover, GLP-2 partially restored the bacteria-fungi interkingdom interaction network of SBS rats.

**Conclusion:**

Our study confirms the bacterial and fungal dysbiosis in type 2 SBS rats, and GLP-2 partially ameliorated these microbiota dysbiosis.

**Supplementary Information:**

The online version contains supplementary material available at 10.1186/s12879-021-06270-w.

## Background

Short bowel syndrome (SBS), a main cause of intestinal failure, refers to a kind of disease presented as severe malnutrition, diarrhea, water and electrolyte disorder mainly resulting from massive small bowel resection [1, 2]. The treatment of SBS is facing multiple challenges. Inadequate enteral nutrition may lead to a series of complications, such as malnutrition, parenteral nutrition related liver injury or catheter-related infection, creating huge economic burdens both for families and the whole society. Gut microbiota dysbiosis is another great challenge for SBS patients [3, 4]. In recent years, with the rapid development of high-throughput sequencing technology, a growing number of studies have been focused on intestinal flora dysbiosis in SBS. Previous studies demonstrated that the intestinal flora of SBS was significantly disturbed, manifested as the reduction of intestinal flora diversity, and increase of pathogenic bacteria abundance. Unfortunately, intestinal flora dysbiosis in SBS can lead to intestinal mucosal inflammation, delayed enteral nutrition and prolonged use of parenteral nutrition, impaired intestinal adaptation, and finally resulting in poor prognosis [4–6]. Our Previous studies have also disclosed that the composition of intestinal flora was altered in type 2 SBS patients and rats, which have an enterocolonic anastomosis and without ileocecal region [2, 7].

Apart from bacteria, intestinal fungi are an indispensable part of intestinal microbiota. However, due to the restriction of techniques in the past, research on intestinal fungi dysbiosis in SBS patients and rats is still lacking. The development of high-throughput technology provides sufficient technical support for the study of fungal microbiota, which enables researchers to accurately understand the composition of intestinal fungi through internal transcribed spacer (ITS) sequencing [8–11]. Recent studies have revealed that there is an intense disruption in intestinal bacteria-fungi correlation network in inflammatory bowel disease (IBD) [8, 9, 11]. In fact, under circumstances of intestinal bacterial dysbiosis, intestinal fungal dysbiosis is likely to occur. Thus, we hypothesized that the fungal microbiota might be altered and further affect the intestinal adaption procedure in SBS.

The intestine is the most developed endocrine organ. There are more than ten kinds of enteric endocrine cells (EEC) in the intestine, which secrete more than twenty kinds of intestinal endocrine hormones [12]. Glucagon like peptide-2 (GLP-2), secreted by distal small intestine and colon, is a well-known intestinal growth factor, which can promote the proliferation of intestinal mucosa and enhance the absorption function of remanent intestine. GLP-2 is the most crucial factor in current intestinal rehabilitation therapy of SBS. Our recent study published in *Theranostics* further demonstrated that GLP-2 exerted its gut mucosal hyperplasia promoting effect through modulating exosomal miRNAs transportation in small intestinal microenvironment [1]. Moreover, Yu Hu et al. [13] declared that GLP-2 significantly alleviated intestinal flora dysbiosis in elderly rats. However, it remains unknown whether GLP-2 could modulate the intestinal bacterial and fungal dysbiosis in SBS. In the current study, we characterized the altered composition of bacteria and fungi in SBS rats and evaluated the effects of GLP-2 on alleviation of bacterial and fungal dysbiosis, and provided insight into the crosstalk between bacteria and fungi in SBS rats.

## Methods

### Animal experimental protocol

**Fig. 1:**
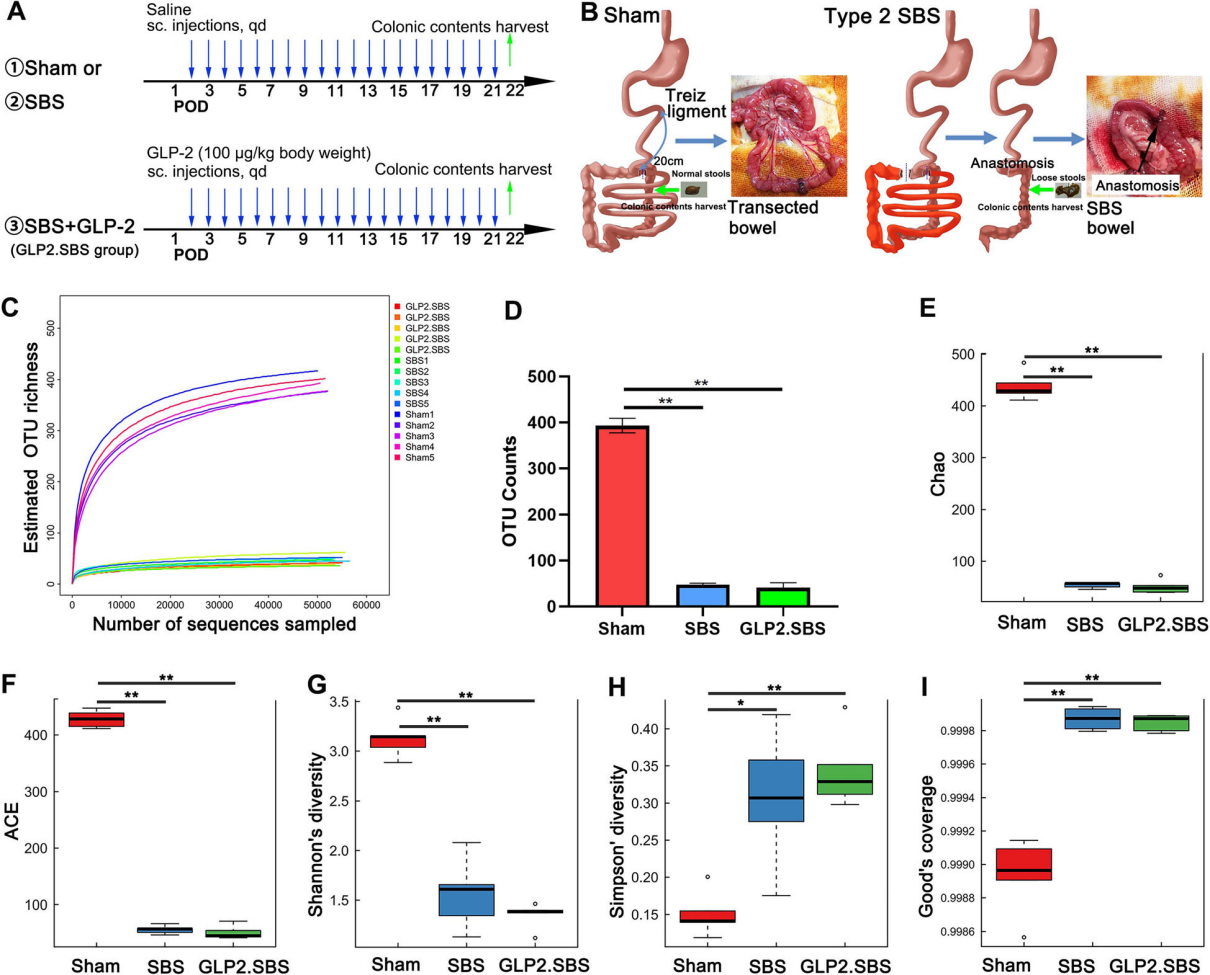
Bacterial microbiota α-diversity biodiversity analysis. **A** Experimental design and procedure of this study. **B** The establishment of sham and type 2 SBS rat model. **C** Rarefaction curves of each sample. **D** OTU counts analysis among groups. Bacterial α-diversity estimated by Chao [**E**], ACE [**F**], Shannon’s diversity [**G**], Simpson’ diversity [**H**] and Good’s coverage [**I**]. D-I Median and IQR were shown. The abnormal value is shown as ‘o’. *P < 0.05; **P < 0.01 compared with the Sham group

Type 2 short bowel syndrome model was created with SD rats (male, 8 weeks old, 250–260 g; Charles River, Beijing, China), according to a previously reported method [1]. Rats were kept at a temperature of 21 °C to 25 °C, humidity of 52 to 62%, and a 12/12-h light/dark cycle. Figure 1A displayed the timeline of the experimental procedures. SD rats were fasted for 24 h before operation with free access to water. All procedures were performed under the anaesthetization of sodium pentobarbital (40 mg/kg, ip) using aseptic technique. Rats were randomly assigned to three experimental groups: Sham, SBS and GLP2.SBS. The Sham group underwent transection and re-anastomosis at approximately 20 cm from the ligament of Treitz without small bowel resection and were given saline as placebo. SBS rats underwent resection of 80% small gut, ileocecum resection, resection of partial colon and jejunocolostomy (shown in Fig. 1B). Animals were resuscitated by intraperitoneal injection of 5 ml saline. 250 ul Salinen or 100 μg/kg body weight degradation-resistant (Gly2) GLP-2 (100 μg/ml dissolved in saline, Creative Peptides, New York, USA) were administered subcutaneously once daily in SBS and GLP2.SBS group, respectively. Rats were fasted within 24 h after operation, then fed with enteral nutrition solution (full strength) for 24–72 h, followed by a gradual transition to solid diet 7 days after operation and maintained total solid diet until the rats were sacrificed for sampling.

### Sample collection and genomics DNA isolation

After study, all animals were anaesthetized by sodium pentobarbital (40 mg/kg, ip) and then euthanized by cervical dislocation at 22 days after operation. At about 3 cm distal to the anastomotic site of SBS rats or the same colon segment site of Sham rats (Fig. 1B), colonic contents were harvested, quickly frozen in liquid nitrogen and stored at − 80 °C for further analysis. The colonic contents DNA was extracted using the QIAamp DNA Stool Mini Kit (Qiagen, Valencia, CA) following the manufacturer’s instructions. DNA was quantified with a Qubit Fluorometer by using Qubit dsDNA BR Assay kit (Invitrogen, USA) and the quality was checked by running aliquot on 1% agarose gel.

### Library construction

The 16S rRNA gene was amplified using primers targeting the V4 region (primers in Table 1). The fungal ITS1 region was amplified using ITS-F and ITS-R primers (in Table 1). Both primers (forward and reverse) were labelled with Illumina adapter. After polymerase chain reaction (PCR), Agencourt AMPure XP beads were used to purify the PCR products. Agilent Technologies 2100 bioanalyzer (Agilent, USA) was used to qualify the libraries. Further, the validated libraries were sequenced on Illumina HiSeq 2500 platform.

**Table 1:** PCR primers

Primer name	Sequences
Bacterial 16S rRNA gene	
515-F	5′-GTGCCAGCMGCCGCGGTAA-3′
806-R	5′-GGACTACHVGGGTWTCTAAT-3′
Fugal ITS1	
ITS-F	5′-CTTGGTCATTTAGAGGAAGTAA-3′
ITS-R	5′-GCTGCGTTCTTCATCGATGC-3′

### Sequencing, bioinformatics and statistical analysis

Paired-end reads were added to tags by FLASH program [14]. Using UPARSE algorithm [15], tags were clustered into operational taxonomical units (OTUs). OTU representative sequences were then taxonomically classified using Ribosomal Database Project Classifier v.2.2, and trained on the UNITE (V6 20,140,910) (for ITS sequencing) and Greengenes database v201305 (for 16 s rRNA sequencing) by QIIME v1.8.0 [16, 17], respectively. Then we used the USEARCH_global to compare all Tags back to OTU.

Alpha and beta diversity were estimated by MOTHUR (v1.31.2) and QIIME (v1.8.0). Sample cluster was conducted by QIIME (v1.8.0). OTU Rank curve was plotted with R package version 3.1.1. The Venn plots in OTUs or in taxa were plotted with R package “Venn Diagram”. Principal Coordinate Analysis (PCoA) was performed by QIIME (v1.8.0). Partial least-squares discrimination analysis (PLS-DA) and Anosim analysis were performed using R package. Heatmap and barplot of different classification levels was plotted with R package “gplots” and R package v3.4.1, respectively. Significant taxa were determined by R package based on Kruskal-Test. LEfSe cluster and LDA analysis were performed by LEfSe (https://huttenhower.sph.harvard.edu/galaxy/). Spearman correlation analysis was applied to build the correlation networks between different bacteria. The correlation networks between top 50 bacteria and top 20 fungi were analyzed using Spearman correlation analysis.

Only differences with adjusted *P*-value of less than 0.05 were considered statistically significant. For data passed Shapiro-Wilk normality test, one-way ANOVA and Tukey’s multiple comparisons test were used, and data were shown as mean (SD or SEM). Otherwise, Kruskal-Wallis and Dunn’s multiple comparisons test were used, and data were shown as median and interquartile range (IQR) using GraphPad Prism 7.

## Results

Colonic contents harvested from 15 rats (5 in each group) were collected and a total of 919,054 V4 16S tags were generated for final analysis, with 61,270 16S tags on average. And 928,933 ITS tags were generated in total with 61,928 tags per sample on average.

### GLP-2 partially reversed intestinal bacterial dysbiosis in SBS rats

Rarefaction analysis demonstrated that the estimated OTU richness could approach saturation in each sample (Fig. 1C). The OTU counts index in SBS and GLP2.SBS group were dramatically lower than that of Sham group, indicating SBS induced a significant decrease in intestinal flora diversity (Fig. 1C-D). Besides, compared with Sham group, the α diversity of intestinal bacteria in SBS rats was significantly decreased, which was manifested as decreased Chao (*P* < 0.01), ACE (*P* < 0.01), Shannon’s diversity (*P* < 0.01), and increased Simpson’ diversity (*P* < 0.05) and Good’s coverage (*P* < 0.01). However, no significant difference in α-diversity was found between SBS and GLP2.SBS group (Fig. 1E-I).

**Fig. 2:**
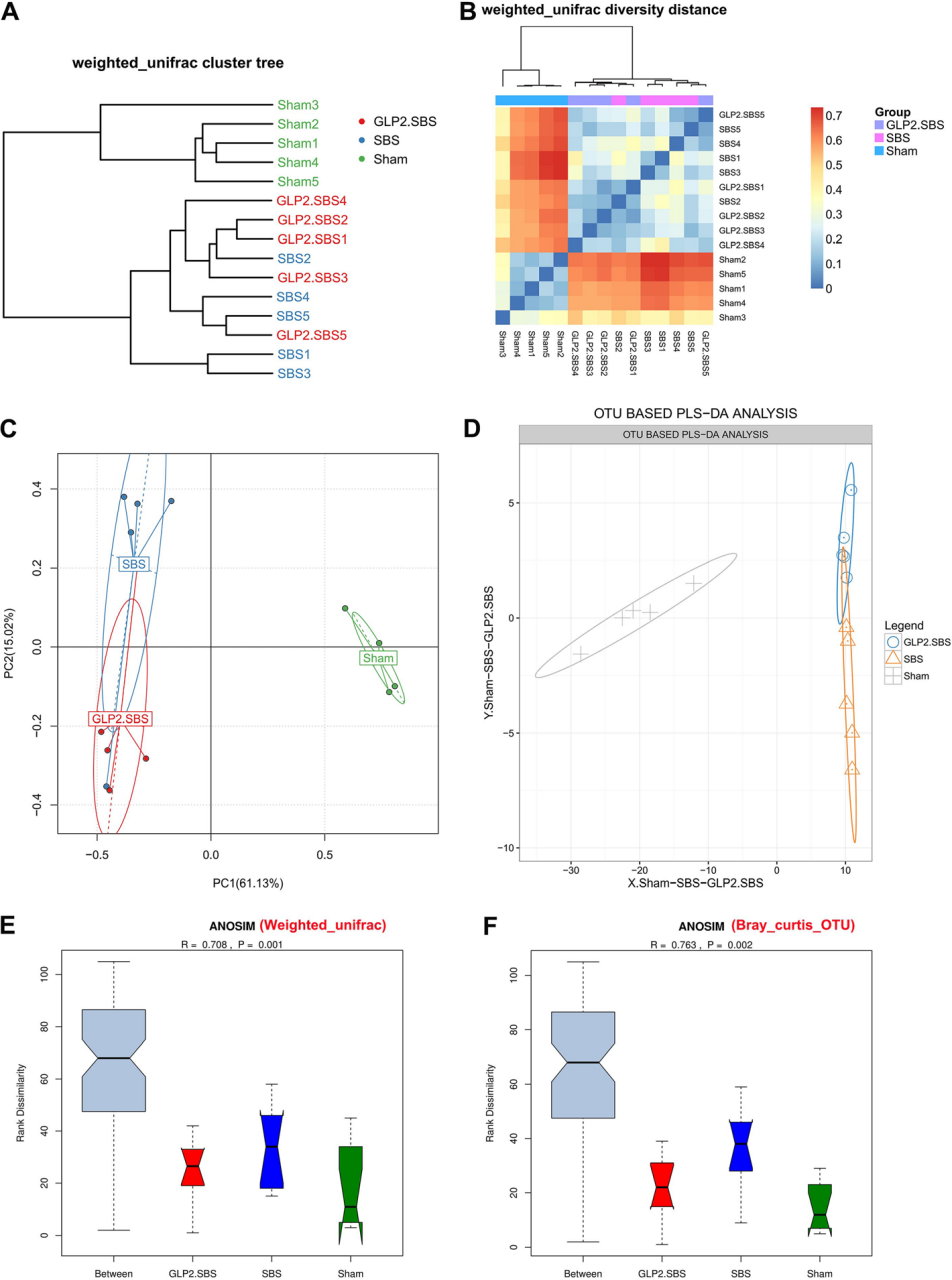
Comparative analyses of the bacterial microbiota communities. **A** Samples’ clustering result (Description, weighted_unifrac). **B** Beta diversity heat map (Description, weighted_unifrac). **C** Bacterial β-diversity of principal coordinates analysis (PCoA) based on weighted_unifrac distance rarefaction curves of each sample. **D** Partial least squares discriminant analysis (PLS-DA) plots based on the level of OTU. **E** Anosim similarity analysis based on weighted_unifrac distance rarefaction curves of each sample. **F** Anosim similarity analysis based on Bray_curtis_OTU distance rarefaction curves of each sample

**Fig. 3:**
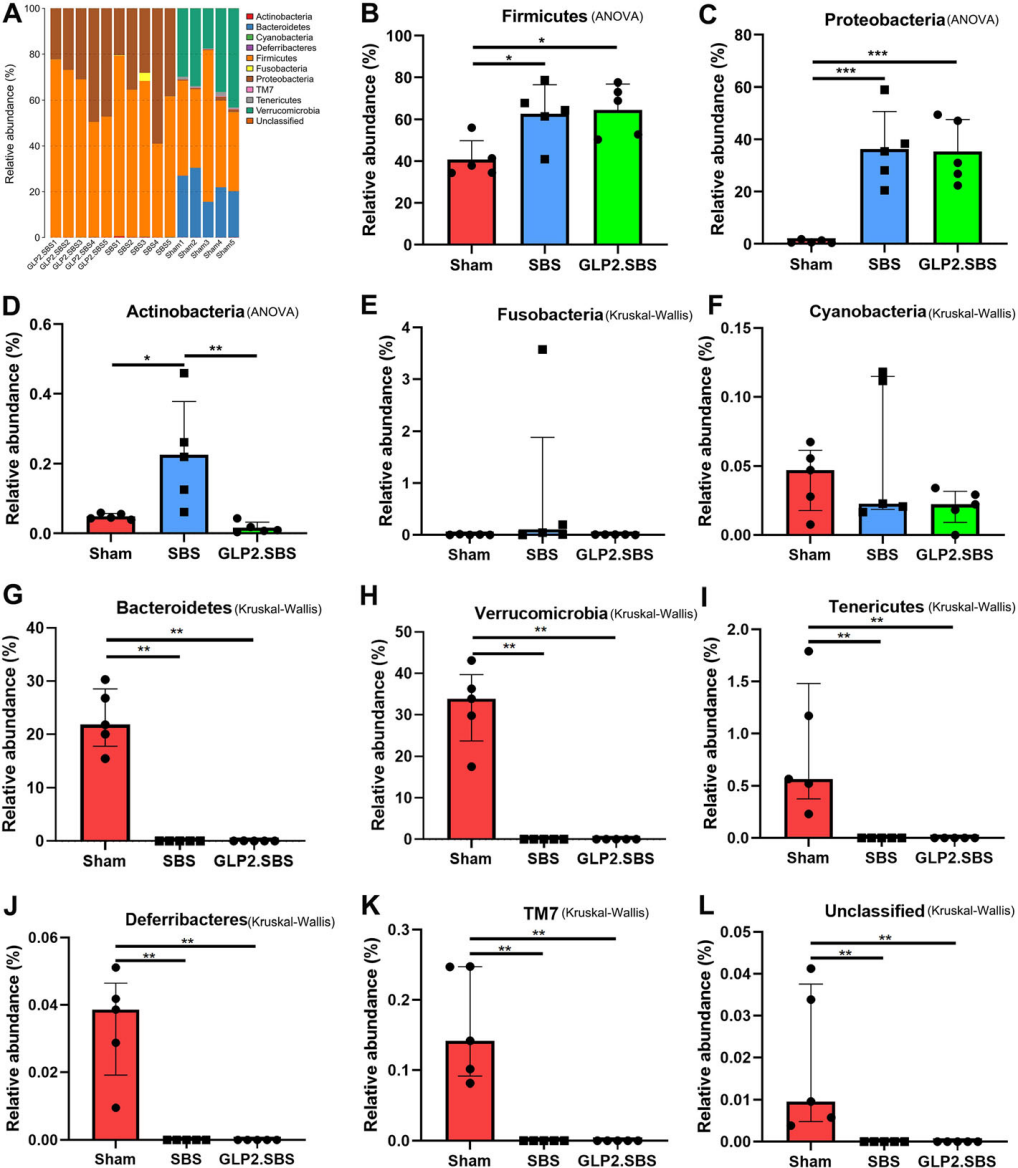
Composition of the bacterial gut microbiota. **A** Proportion of different phyla in each sample. **B**-**L** Relative abundance of top 11 phylum across groups. **B**-**D** One-way ANOVA and Tukey’s multiple comparisons test were used, and data were shown as mean ± SD; **E**-**L** Kruskal-Wallis and Dunn’s multiple comparisons test were used, and data were shown as median and IQR. *P < 0.05; **P < 0.01; ***P < 0.001

Beta diversity, assessed by the weighted UniFrac Cluster Tree and weighted UniFrac diversity distance, were different in both SBS groups as compared with the Sham group (Fig. 2A, B). We then visualized weighted UniFrac dissimilarity by PCoA analysis (PC1 61.13% and PC2 15.02%), which declared different overall bacterial community among Sham, SBS and GLP2.SBS group. We found the samples of Sham group clustered together and were far away from the fusion clustering of SBS group and GLP2.SBS group (Fig. 2C). Additionally, PLS-DA, a supervised analysis method also disclosed a distinctive intestinal bacterial composition in GLP2.SBS and SBS groups when compared with the Sham group (Fig. 2D). Furthermore, Anosim similarity analysis based on both weighted_unifrac and Bray_curtis_OTU distance disclosed a statistically different intestinal bacterial composition between Sham group and the two SBS groups (SBS group and GLP2.SBS group) (Fig. 2E, F and Figure S1). The colonic flora composition differed dramatically at phylum level among the three groups (Fig. 3A). Analyzed at phylum level, rats underwent massive small bowel resection displayed perturbed microbiota signatures especially in Firmicutes, Proteobacteria, Actinobacteria, Bacteroidetes, Verrucomicrobia, Tenericutes, Deferribacteres, TM7 and Unclassified phyla compared with Sham rats (Fig. 3B-L). In SBS and GLP2.SBS group, Firmicutes dramatically increased as compared with Sham group (*P* = 0.0320 and *P* = 0.0204) (Fig. 3B). Meanwhile, the relative abundance of Proteobacteria was quite low in Sham group, while it was significantly higher in the colonic contents of SBS and GLP2.SBS group. Furthermore, compared to Sham group, Actinobacteria was significantly increased in SBS group, while GLP-2 treatment decreased the relative abundance of Actinobacteria in SBS rats (Fig. 3D). The relative abundance of Bacteroidetes, Verrucomicrobia, Tenerictes, Deferrribates, TM7 and Unclassified phyla were significantly decreased, even undetectable in SBS and GLP2.SBS rats, indicating that the intestinal bacterial flora of SBS rats has been seriously disturbed (Fig. 3G-L).

**Fig. 4:**
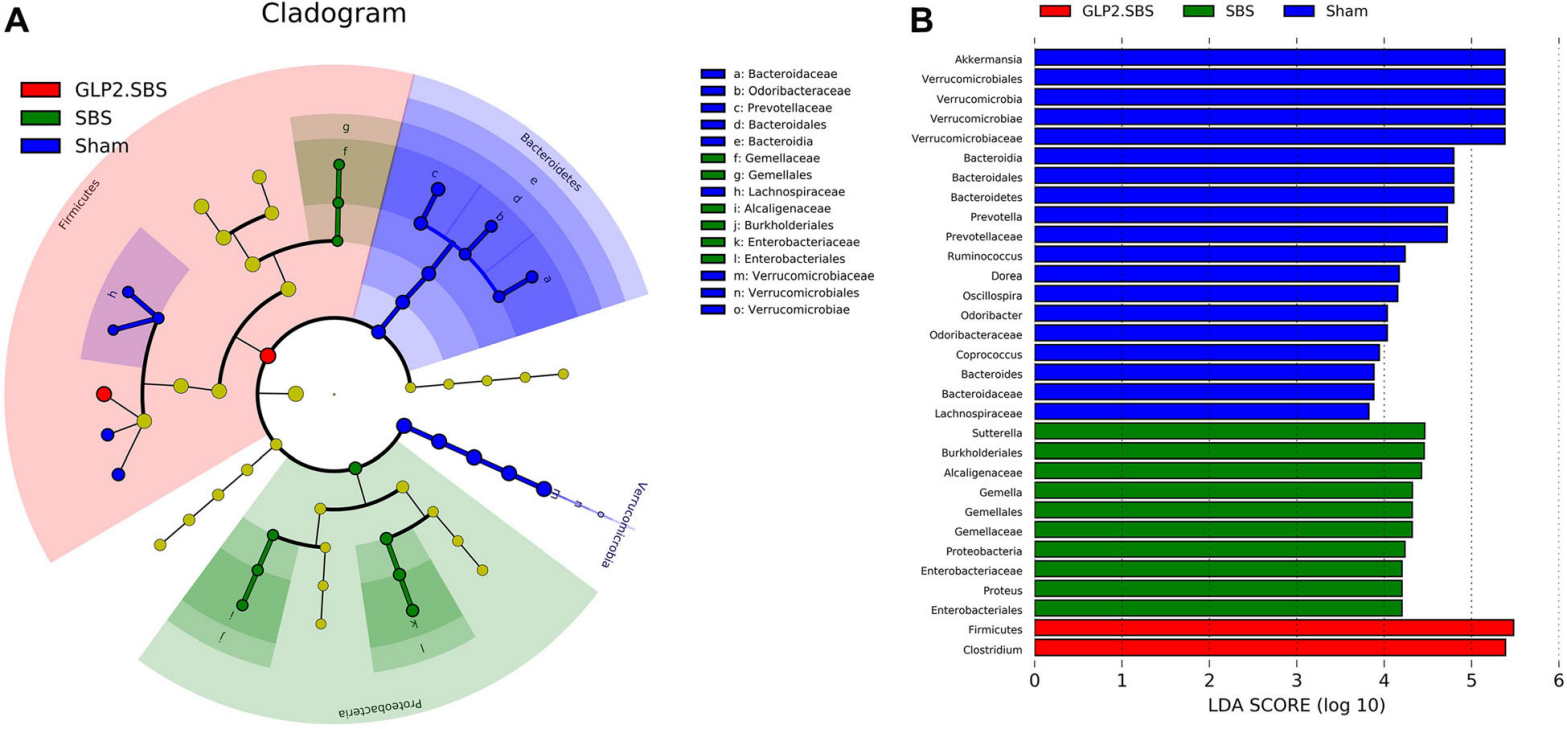
Variations of the bacterial gut microbiota. **A** Cladogram depicting the taxonomic hierarchical structure of distinguished phylotype created by LEfSe analysis. Each filled circle indicates a certain type of phylotype. Different colors represent different groups. Colored node consistent with the group color indicated important microbe biomarkers in the group and the name of biomarkers are listed in the upper right corner. The yellow notes suggest biomarkers which do not show any significant differences among groups. **B** Graphics of Linear discriminant analysis (LDA). Horizontal bars demonstrated the effect size. The length of the bar indicates the log_10_ transformed LDA score, represented by vertical dotted lines. The bacteria with statistically significant changes (*p* < 0.05) are shown alongside the horizontal lines

We further analyzed the different bacterial strains of the three surgical groups and the subordination of these different bacterial strains by LEfSe. SBS resulted in an obvious reduction of multiple bacterial genera, including Bacteroides, Odoribacter and Prevotella genera from Bacteroidetes phylum, Coprococcus and Dorea genera from Lachnospiraceae family and Firmicutes phylum, Oscillospira, Ruminococcus and Clostridium genera from Ruminococcaceae family and Firmicutes phylum, and Akkermansia genus from Verrucomicrobia phylum. Moreover, SBS caused a significant increase of Proteus genus belonging to the pro-inflammatory Proteobacteria phylum. On the other hand, GLP-2 treatment could partially ameliorate the intestinal bacterial dysbiosis of SBS rats, manifested as significant inhibition of the overgrowth of pro-inflammatory Proteus genus in SBS rats. Moreover, the relative abundance of anti-inflammatory Clostridium genus from Firmicutes phylum was remarkably increased in GLP2.SBS rats when compared with SBS rats (Fig. 4 and Table 2).

**Table 2:** Relative abundance of bacteria of colonic samples

Bacterial taxa	Sham	SBS	GLP2.SBS
Bacteroidetes.P			
Bacteroides.G	0.55 (0.42–1.12)	0.00 (0.00–0.01) ^*^	0.00 (0.00–0.00) ^**^
Odoribacter.G	0.75 (0.48–1.15)	0.00 (0.00–0.00) ^**^	0.00 (0.00–0.00) ^**^
Prevotella.G	6.40 (4.63–8.32)	0.00 (0.00–0.00) ^**^	0.00 (0.00–0.02) ^*^
Firmicutes.P			
Lachnospiraceae.F	3.35 (2.49–4.01)	0.00 (0.00–0.21) ^*^	0.02 (0.00–0.20) ^*^
Coprococcus.G	0.44 (0.23–0.63)	0.00 (0.00–0.00) ^**^	0.00 (0.00–0.00) ^**^
Dorea.G	0.04 (0.02–0.36)	0.00 (0.00–0.00) ^**^	0.0000 (0.00–0.00) ^**^
Ruminococcaceae.F	5.73 (4.48–7.34)	0.00 (0.00–0.00) ^*^	0.00 (0.00–0.00) ^*^
Oscillospira.G	1.55 (1.23–1.98)	0.00 (0.00) ^**^	0.00 (0.00) ^*^
Ruminococcus.G	1.70 (1.29–2.34)	0.00 (0.00–0.00) ^*^	0.00 (0.00–0.00) ^*^
Clostridium.G	0.09 (0.05)	15.10 (11.40) ^**^	33.55 (12.67) ^***#^
Verrucomicrobia.P			
Akkermansia.G	33.90 (23.69–39.75)	0.00 (0.00–0.00) ^*^	0.00 (0.00–0.00) ^*^
Proteobacteria.P			
Proteus.G	0.00 (0.00–0.00)	2.01 (1.23–2.37) ^**^	0.02 (0.02–0.05) ^#^

Data were expressed as percentages of total sequences. For data passed normality test, one-way ANOVA and Tukey’s multiple comparisons test were used, and data were shown as mean (SD). Otherwise, Kruskal-Wallis and Dunn’s multiple comparisons test were used, and data were shown as median and IQR

.*P* Phylum, *F* Family, *G* Genus

*P < 0.05; **P < 0.01; ***P < 0.001 compared with Sham group; ^#^*P* < 0.05 compared with SBS group

**Fig. 5:**
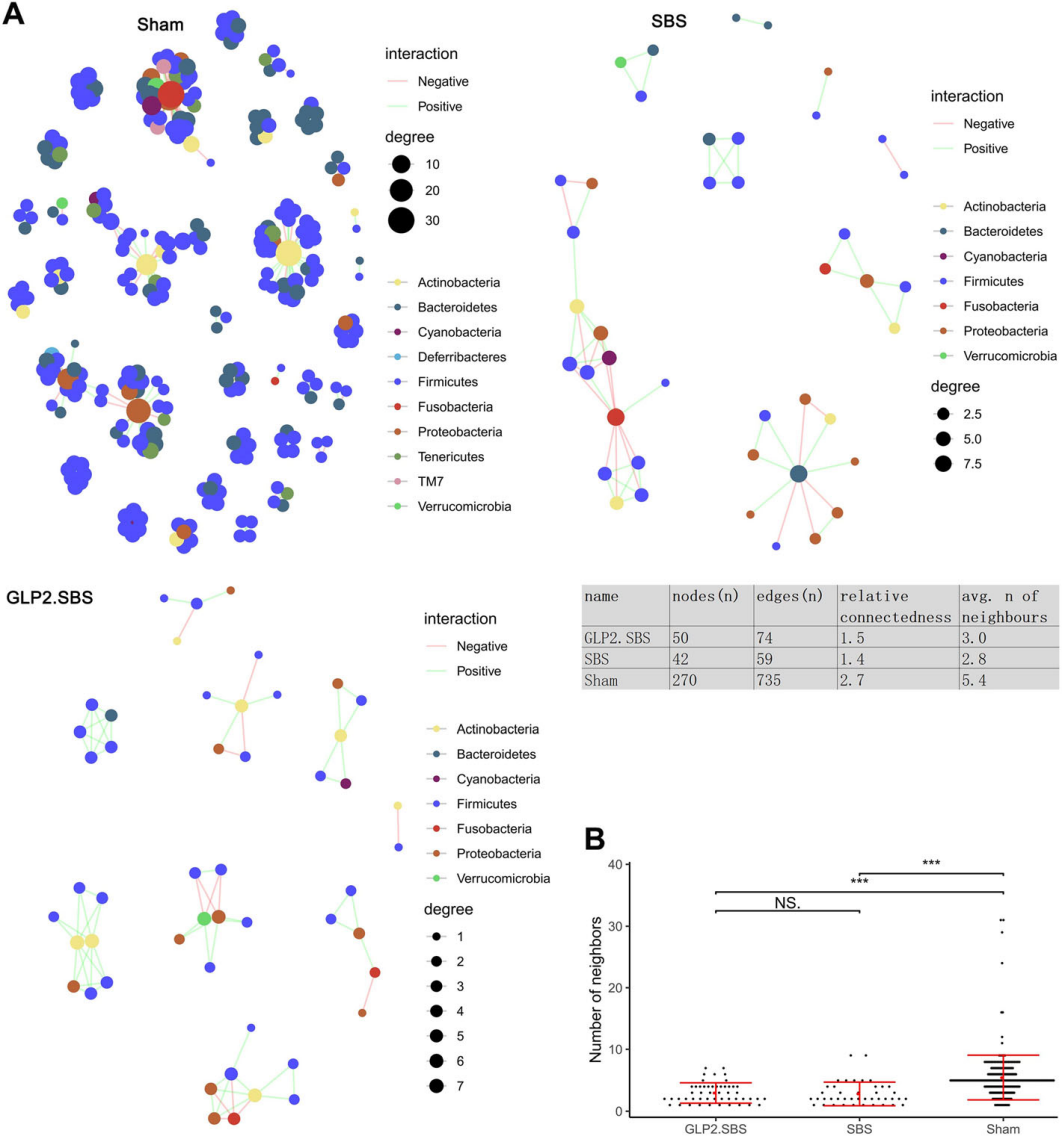
Correlation networks of intestinal bacterial community. Correlations between bacteria were analyzed by Spearman correlation analysis. **A** Correlation networks of abundance are shown, in which each node represents an OTU, its color represents the bacterial phylum and its size indicates the number of direct edges. Edges suggest the magnitude of distance correlation (positive in green, negative in red). Only OTUs presented in > 1/3 of all samples would be taken into account. The table in the middle panel shows the parameters of network. The relative connectivity of networks was calculated as the ratio of the number of significant interactions to the number of nodes in the correlation network. **B** Quantification of neighbor amount in Sham, SBS and GLP2.SBS; means and SEM are marked in red. ***p < 0.001. avg., average

We further built the bacterial abundance correlation network to assess the bacterial structure in SBS rats. An abundant and complicated network of interrelations between bacteria was observed in Sham rats (Fig. 5A). In comparison with the Sham rats, the density of bacterial interrelation network was markedly decreased in SBS rats, as attested by a reduced relative connectedness and a decreased number of neighbors. The network in GLP2.SBS rats was larger (nodes *n* = 50, edges *n* = 74) than in SBS rats (nodes *n* = 42, edges *n* = 59), suggesting a potential ameliorate effect of GLP-2 treatment (Fig. 5A, B).

### GLP-2 ameliorated intestinal fungal dysbiosis in SBS rats

**Fig. 6:**
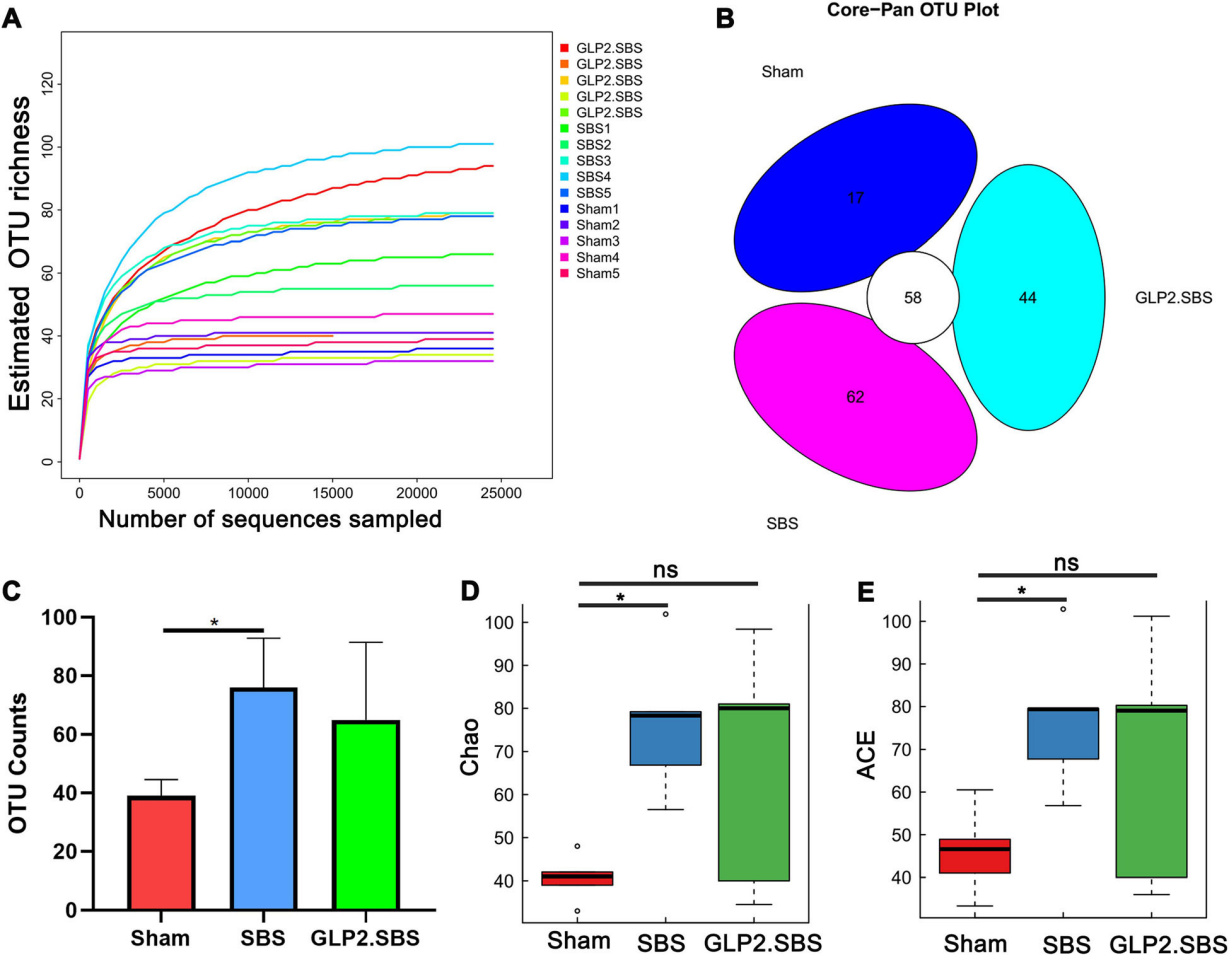
Fungal microbiota α-diversity biodiversity analysis. **A** Rarefaction curves of each sample. **B** Core-Pan OTU analysis, circle in the middle indicates the number of shared OTUs in three groups, and the ellipse outside the middle circle suggest the number of OTUs unique to certain group. **C** OTU counts analysis among groups. Fungal α-diversity estimated by Chao [**D**] and ACE index [**E**]. **C** One-way ANOVA and Tukey’s multiple comparisons test were used, and data were shown as mean ± SD; **D**-**E** Kruskal-Wallis and Dunn’s multiple comparisons test were used, and data were shown as median and IQR. The abnormal value is shown as ‘o’. *P < 0.05 compared with the Sham group

Intestinal bacterial dysbiosis could result in intestinal fungal dysbiosis, but there is still no research on intestinal fungal dysbiosis in SBS rats and patients [3]. This part aims to investigate the intestinal fungal dysbiosis of SBS and the therapeutic effect of GLP-2 on the intestinal fungal dysbiosis. Rarefaction analysis declared that the estimated OTU richness could approach saturation in each sample (Fig. 6A). OTU Venn analysis showed that there were 58 mutual OTUs in the three groups. And there were 17 OTUs, 62 OTUs and 44 OTUs specifically in Sham group, SBS group and GLP2.SBS group (Fig. 6B). Moreover, statistics showed that there were significant differences in OTU Counts index between Sham group and SBS group (*P* < 0.05). On the other hand, GLP-2 treatment inhibited the increase of OTU Counts index, manifested by no significant difference in OTU Counts index between Sham group and GLP2.SBS (Fig. 6C). Moreover, the α-diversity of intestinal fungi in SBS rats was dramatically increased relative to Sham rats, as evidenced by increased Chao (*P* < 0.05) and ACE index (*P* < 0.05). As expected, no significant difference in Chao and ACE index was found between GLP2.SBS group and Sham group, indicating that GLP-2 treatment might partially reverse the intestinal fungal overgrowth in SBS rats (Fig. 6D-E).

**Fig. 7:**
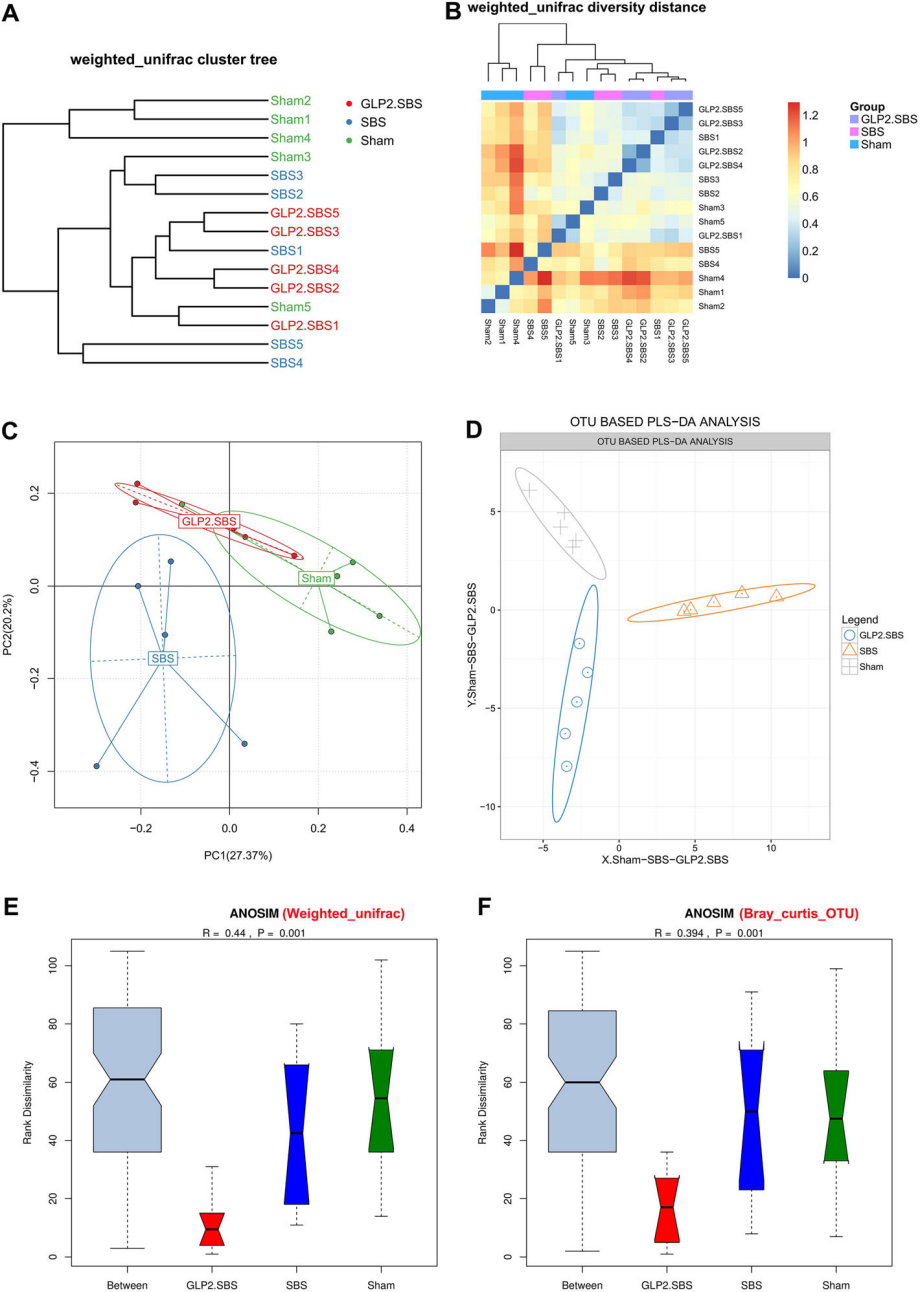
Comparative analyses of intestinal fungal microbiota communities. **A** Samples’ clustering result (Description, weighted_unifrac). **B** Beta diversity heat map (Description, weighted_unifrac). **C** Fungal β-diversity of PCoA based on weighted_unifrac distance rarefaction curves of each sample. **D** PLS-DA plots based on the level of OTU. **E** Anosim similarity analysis based on weighted_unifrac distance rarefaction curves of each sample. **F** Anosim similarity analysis based on Bray_curtis_OTU distance rarefaction curves of each sample

Weighted UniFrac Cluster Tree and weighted UniFrac diversity distance showed obvious changes in intestinal fungal β-diversity in SBS and GLP2.SBS rats when compared with Sham rats (Fig. 7A, B). We then visualized UniFrac dissimilarity by PCoA analysis, which indicated different overall fungal community structures among Sham, SBS and GLP2.SBS group. Moreover, we found samples of SBS group clustered together and were far away from the fusion clustering of Sham group and GLP2.SBS group, indicating that GLP-2 treatment could partially reverse the intestinal fungal dysbiosis of SBS rats (Fig. 7C). Additionally, the result of PLS-DA was similar with PCoA analysis result (Fig. 7D). Furthermore, Anosim similarity analysis based on both weighted_unifrac and Bray_curtis_OTU distance disclosed a statistically different intestinal fungal composition between the three groups (Fig. 7E, F and Figure S2).

**Fig. 8:**
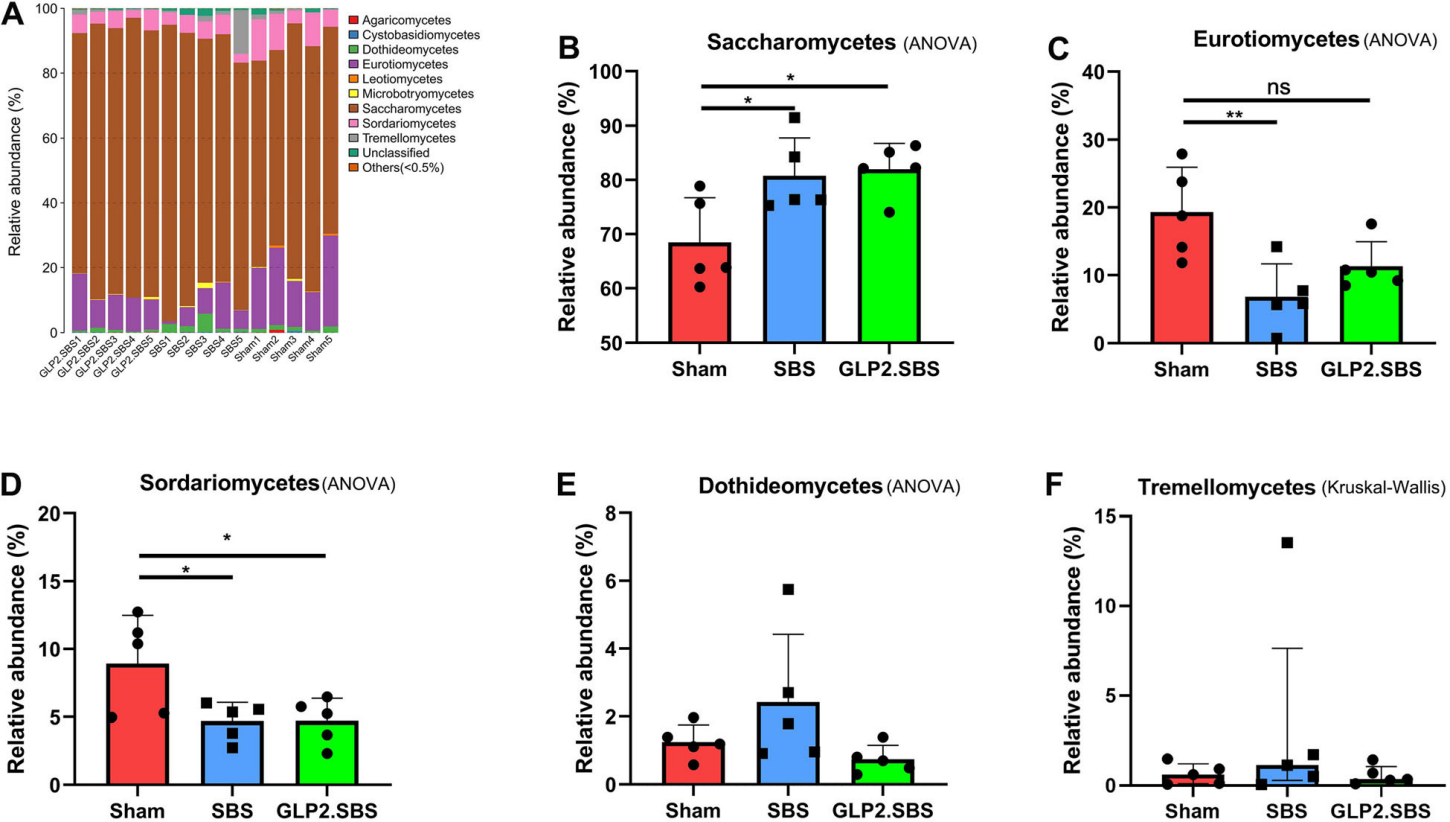
Composition of intestinal fungal microbiota. **A** Proportion of the different classes in each sample. **B**-**F** Relative abundance of top 5 classes of gut fungus among three groups. **B**-**E** One-way ANOVA and Tukey’s multiple comparisons test were used, and data were shown as mean ± SD; **F** Kruskal-Wallis and Dunn’s multiple comparisons test were used, and data were shown as median and IQR. *P < 0.05; **P < 0.01

Figure 8A showed profiling of each sample at the class level and gave an idea of the proportion of different classes in each sample. Furthermore, the differences between groups in the first five classes, including Saccharomycetes, Eurotiomycetes, Sordariomycetes, Dothideomycetes, and Tremellomycetes were investigated. The results suggested that the relative abundance of Saccharomycetes class was notably increased in SBS and GLP2.SBS group (*P* = 0.0367 and *P* = 0.0222) (Fig. 8B). In comparison with Sham group, the relative abundance of Eurotiomycetes class was remarkably reduced in SBS group (*P* = 0.0067), while GLP-2 treatment partially reversed the decrease of Eurotiomycetes class in SBS rats (Fig. 8C). The relative abundance of Sordariomycetes decreased evidently in both SBS and GLP2.SBS group (*P* = 0.0418 and *P* = 0.0421) (Fig. 8D). The relative abundance of Dothideomycetesand Tremellomycetes in SBS rats was higher than that of rats in Sham group, but there was no significant difference between the three groups (Fig. 8E, F).

**Fig. 9:**
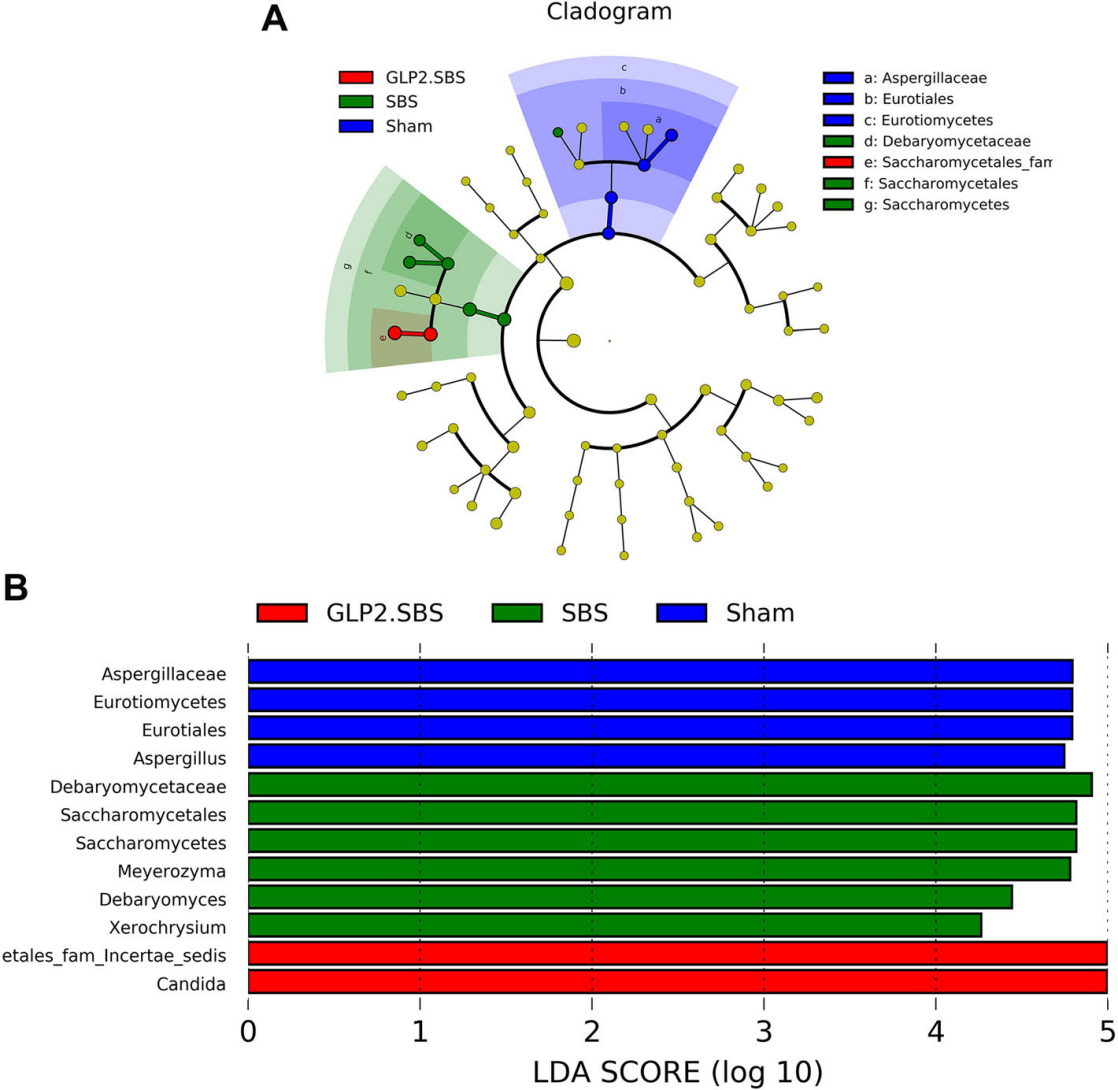
Variations of the fungal gut microbiota. **A** Taxonomic Cladogram depicting the hierarchical structure of distinguished phylotype created by LEfSe analysis. **B** LDA score computed by LEfSe analysis. The fungus with statistically significant changes (*p* < 0.05) is shown alongside the horizontal lines

We then analyzed the different fungal strains in three surgical groups by LEfSe analysis. SBS resulted in a remarkable reduction in the relative abundance of the multiple fungal genera, including Aspergillus and Penicillium genera from Eurotiomycetes class and Wickerhamomyces genus from Saccharomycetes class. Moreover, SBS caused a significant increase in three genera, including Debaryomyces and Meyerozyma genera from Saccharomycetes class and Xerochrysium genus from Eurotiomycetes class. On the other hand, GLP-2 treatment (GLP2.SBS group) can partially ameliorate the intestinal fungal dysbiosis of SBS rats, including significantly inhibited the increase of Meyerozyma and Debaryomyces genera, and significantly inhibited the decrease of Penicillium genera (Fig. 9 and Table 3).

**Table 3:** Relative abundance of fungal genera of colonic samples

Fungal taxa	Sham	SBS	GLP2.SBS
Eurotiomycetes.C			
Aspergillus.G	16.43 (6.48)	5.36 (1.61) ^**^	9.14 (2.99)
Penicillium.G	2.46 (1.07)	0.41 (0.16) ^**^	1.75 (0.65) ^#^
Xerochrysium.G	0.00 (0.00–0.03)	0.31 (0.10–0.75) ^*^	0.11 (0.03–0.50)
Saccharomycetes.C			
Wickerhamomyces.G	7.41 (2.27)	3.33 (1.62) ^*^	4.12 (1.63)
Debaryomyces.G	0.00 (0.00–0.17)	3.90 (2.43–7.56) ^**^	0.30 (0.17–0.37) ^#^
Meyerozyma.G	4.66 (1.51)	13.51 (5.27) ^**^	1.48 (0.26) ^###^

Data were expressed as percentages of total sequences. For data passed normality test, one-way ANOVA and Tukey’s multiple comparisons test were used, and data were shown as mean (SD). Otherwise, Kruskal-Wallis and Dunn’s multiple comparisons test were used, and data were shown as median and IQR

.*C* Class, *G* Genus

*P < 0.05; **P < 0.01 compared with Sham group; ^#^*P* < 0.05; ^###^*P* < 0.001 compared with SBS group

### Altered interkingdom network in SBS rats

**Fig. 10:**
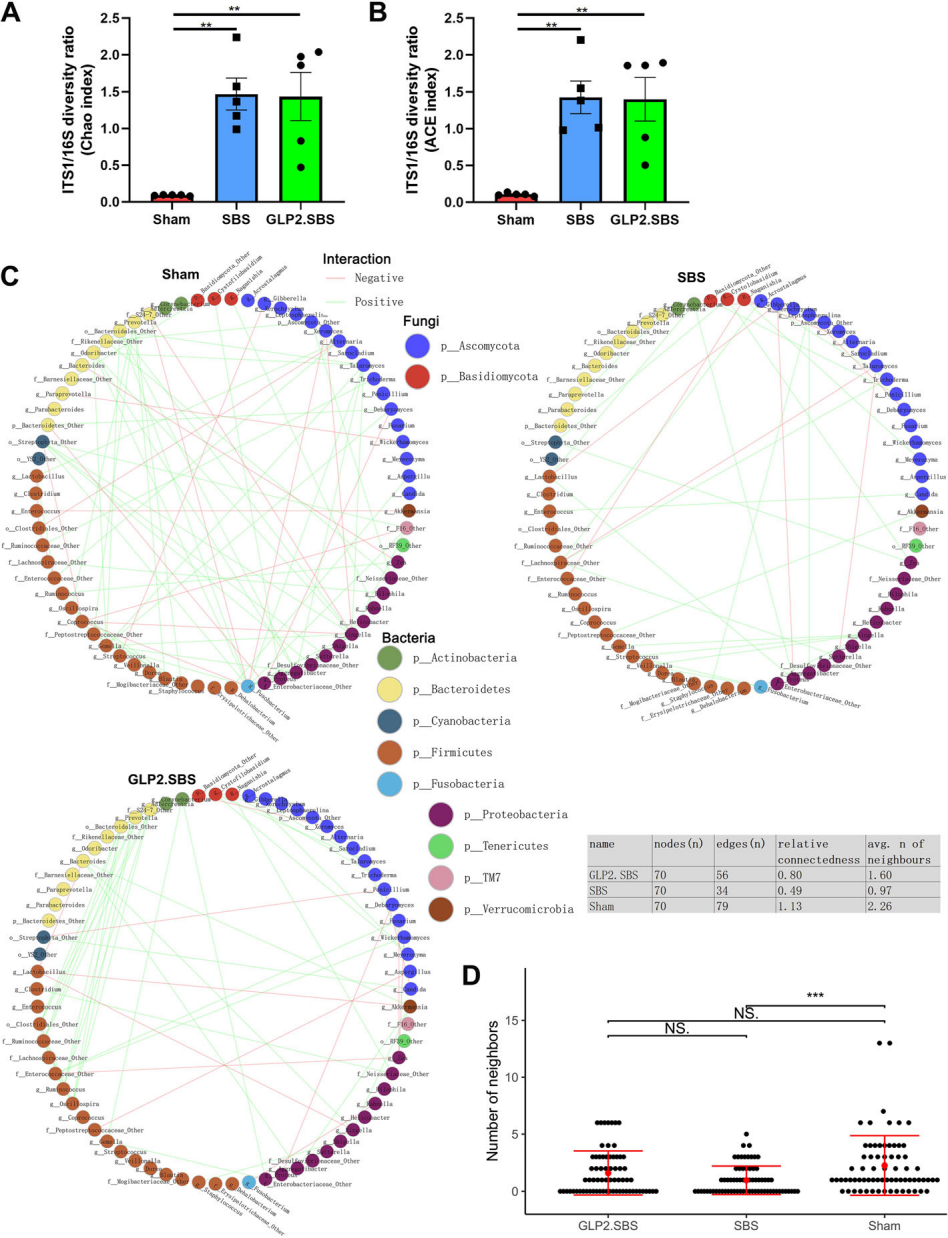
Microbial interrelation network of SBS rats in the validation study. ITS1/16S diversity ratio was computed by the Chao [**A**] and ACE index [**B**] in the three surgical groups; One-way ANOVA test followed by Tukey’s multiple comparisons test were applied to determine differences among groups. **C** The abundance of top 50 bacteria and top 20 fungi was analyzed using Spearman correlation analysis. Only significant correlations [*P* ≤ 0.05] are displayed with an edge. The colors of edge denote positive correlation in green and negative correlation in red. Each node represents certain microbial genera and each color indicates specific microbial phyla. The table shows the parameters of network. The relative connectivity of networks was calculated as the ratio of number of significant interactions [edges] to the number of taxa [nodes] in the network. **D** Average number of neighbors. Means and SEM are indicated in figure [**A**], [**B**] and [**D**]. *p < 0.05; ***p < 0.001. avg., average

The bacteria–fungi interaction has been proven to be quite important for keeping intestinal microbial homeostasis [11, 18]. To investigate the equilibrium between intestinal bacteria and fungi diversity, we calculated the fungi-to-bacteria diversity by ITS1/16S diversity ratio of Chao index and ACE index. The ratios were significantly higher in SBS and GLP2.SBS rats than those in Sham rats. However, there was no significant difference of fungi-to-bacteria diversity ratio between SBS and GLP2.SBS rats (Fig. 10A, B). Furthermore, to explore bacterial and fungi interaction network in this study, we selected the top 20 fungal genera and top 50 bacterial genera for further analysis according to the microbial relative abundance. When compared with Sham group, the correlation between microbiota was quite weak (only few lines between fungal and bacterial genera) in SBS group, indicating the interkingdom network was seriously disrupted. The microbial correlation pattern was stronger in GLP2.SBS group relative to SBS group, indicating by increased edges, average number of neighbors, and relative connectedness (Fig. 10C, D). These data suggested that SBS led to an alteration of bacterial-fungi interactions, whereas GLP-2 treatment could help to partially rebuild these interkingdom interactions.

## Discussion

In the present study, we comprehensively described the intestinal bacterial and fungal dysbiosis in type 2 SBS rats with ileocecal resection, and found that GLP-2 treatment could partially attenuate the disturbance of intestinal microbiota. Although our previous studies have disclosed severe intestinal bacterial dysbiosis in animal models and patients with ileocecal resection [2, 7], there is still no comprehensive assessment of intestinal fungal dysbiosis accompanied by intestinal bacterial dysbiosis in SBS. Due to the interkingdom interaction between bacteria and fungi, intestinal bacterial dysbiosis is likely to cause intestinal fungal dysbiosis, which may further aggravate the intestinal bacterial dysbiosis, and even exacerbate the intestinal inflammation and thus worsen the prognosis of the disease [19–21]. Limited by previous technology, it was difficult for traditional culture-dependent methods to achieve in-depth understanding of the fungal microbiota. Recently, high-throughput sequencing provides effective technical support for study of intestinal fungi in various diseases [20, 22]. Herein, we used high-throughput ITS sequencing to comprehensively analyze intestinal fungal dysbiosis in type 2 SBS rats. Our previous study found that GLP-2 could promote intestinal mucosal proliferation [1]. However, whether GLP-2 could ameliorate the intestinal microbiota dysbiosis of SBS has not been studied. Yu Hu and colleagues [13] demonstrated that GLP-2 treatment resulted in the increase of several probiotics and the decrease of some pathogenic bacterial genera in intestine of aged rats. Therefore, we performed this study to investigate the colonic microbiota of SBS rats with or without GLP-2 intervention.

We first characterized the altered composition of intestinal bacterial dysbiosis in SBS rats and evaluated the therapeutic effects of GLP-2 on intestinal bacteria dysbiosis. Significant decrease of OTU counts and α diversity were observed in the colonic contents of SBS and GLP2.SBS rats which revealed that there was significant intestinal bacterial dysbiosis in type 2 SBS rats. Massive resection of small intestine and ileocecal resection lead to fast pass of food, gastric acid and bile acid, which exerts deleterious effect on the luminal microenvironment. This will inevitably affect the living environment of intestinal microbiota, thus leading to intestinal microbiota dysbiosis, presented as reduction of probiotics and an increase of some pathogenic bacteria [23]. Although GLP-2 possess proliferative and anti-inflammatory effects on intestinal mucosa, no significant improvement in OTU counts and α-diversity was found in GLP-2 treated SBS rats. Both PLS-DA and PCoA strongly indicated distinguished bacterial communities between Sham and SBS group, while clusters of SBS and GLP2.SBS group were close to each other, indicating similar bacterial communities. Moreover, notable differences in β-diversity based on weighted UniFrac analysis and Anosim tests suggested that the variation of microbial community contains not only changes in quantity of bacterial species but also changes in specific bacterial abundance [24]. These results demonstrated that massive resection of small intestine and ileocecal resection could severely disrupt normal intestinal bacterial microbiome, while GLP-2 treatment could not restore the intestinal bacterial diversity of SBS rats.

Detailed analysis of the composition and structure of intestinal bacteria in SBS demonstrated that, in comparison with Sham group, the relative abundance of 9 out of 11 phyla changed significantly, suggesting a thorough dysbiosis of intestinal bacteria in SBS rats. In line with previous studies, there existed a dramatic overabundance of Proteobacteria and its family Enterobacteriaceae in colonic contents of SBS rats, which contains a great number of pathogenic bacteria [2, 25, 26].

Proteus, an opportunistic pathogen within the Enterobacteriaceae family, was remarkably prevalent in SBS group in our study. Previous studies have proved Proteus’ potential ability to produce LPS results in the upregulated secretion of pro-inflammatory cytokines and further leads to the destruction of intestinal mucosal barrier and thus inducing bacterial translocation [2, 27]. A remarkable alteration of the Firmicutes phylum in SBS and GLP2.SBS rats was the decrease of the Lachnospiraceae and Ruminococcaceae families, which are able to produce short-chain fatty acids (SCFAs). SCFAs, important metabolites of bacteria, exerted many important biological functions, such as energy-supplying fuel for intestinal epithelial cells, inducing enterocyte proliferation and differentiation, and promoting the production of antimicrobial peptides by epithelial cells [2, 28, 29]. Therefore, deficiency of Lachnospiraceae and Ruminococcaceae families may have adverse effects on intestinal adaptation of SBS. In line with previous research, the relative abundance of Akkermansia genus from Verrucomicrobia phylum decreased significantly and even disappeared completely in SBS rats [2]. Akkermansia is a newly discovered star probiotics, which has a variety of beneficial biological functions, such as anti-tumor effect, intestinal mucosal barrier protection, and alleviating colonic inflammation [30–32]. Yu Xu and colleagues summarized that Akkermansia could protect the intestinal mucosal barrier and inhibit the translocation of intestinal flora, therefore reducing the level of LPS in circulation and thus inhibiting the inflammatory response [33]. The almost complete loss of Akkermansia in the colon caused by SBS might affect the integrity of the intestinal mucosal barrier of SBS, lead to bacterial translocation and aggravate the systemic inflammatory response, which may have adverse effects on the intestinal adaptation of SBS.

However, effective therapies for intestinal bacterial dysbiosis are still lacking. The first drug we thought of and used was antibiotics. However, a previous study had found that several days of antibiotics may lead to a reduction of potentially anti-inflammatory Clostridia and an increase of pro-inflammatory Proteobacteria [4]. According to previous studies, GLP-2 may alleviate intestinal microbiota dysbiosis [13]. As we expected, GLP-2 treatment significantly downregulated the relative abundance of pro-inflammatory Proteus genus in SBS rats, and increased the relative abundance of potentially anti-inflammatory inhibitory Clostridium genus from Clostridia class in SBS rats. Notably, some Clostridium spp. could also be unbeneficial, and were even increased in patients with inflammatory bowel disease when compared with healthy people [34].

As we expected, the colonic fungi of SBS rats were abnormal, indicated by increased OTU Counts index, Chao and ACE index. There was obvious overlap between the PCoA cluster of Sham group and the GLP2.SBS group, suggesting that their fungal communities were similar. On the contrary, there was no overlap between the PCoA clusters of Sham group and SBS group, suggesting that their fungal structures were quite different. Therefore, GLP-2 treatment not only remarkably inhibited the abnormal reproduction of colonic fungi, but also potentially improved the SBS colonic fungal communities, making it partially restored to the Sham rats. Kelly B. Flett and Hector Chavez [35, 36] demonstrated a high proportion of blood fungal infections in SBS patients using culture-dependent methods, but did not use high-throughput sequencing to analyze intestinal fungal dysbiosis. To our knowledge, our study comprehensively analyzed the fungal dysbiosis in SBS by high-throughput sequencing for the first time, and the results will provide preliminary data support for relevant studies on SBS fungal dysbiosis.

In terms of phyla, we found the relative abundance of Ascomycota phylum in Sham, SBS and GLP2.SBS was over 90%, indicating Ascomycota was the most predominant phylum in the intestine, followed by Basidiomycota phylum. This result is consistent with many previous studies [8, 9, 37]. Furthermore, we comprehensively analyzed the composition of colonic fungi at the class level. The results showed that 3/5 of the top five abundant fungi classes (including Saccharomycetes, Eurotiomycetes, Sordariomycetes) were significantly different in SBS rats in comparison with the Sham rats. Though the other 2/5 classes (including Tremellomycetes, Dothideomycetes) were increased in SBS rats, they did not reach significant difference (probably because of the small sample size). These results indicated that severe intestinal fungal dysbiosis occurred in SBS rats. LEfSe analysis was used to deeply analyze the different fungi in the 3 groups, and some interesting results were found. For example, the relative abundance of Penicillium genus from Eurotiomycetes class decreased significantly in the SBS rats, but GLP-2 treatment could reverse its decrease in SBS rats. In addition, the abundance of Debaryomyces and Meyerozyma genera from Saccharomycetes class increased significantly in the SBS group, while GLP-2 treatment could inhibit its overgrowth. In fact, at the genus level, SBS group exhibited significant changes in six fungal genera (including Aspergillus, Penicillium, Xerochrysium, Wickerhamomyces, Debaryomyces and Meyerozyma) as compared with Sham group, while GLP-2 treatment could partially reverse the significant changes of 3/6 fungal genera (including Penicillium, Debaryomyces and Meyerozyma) at 22nd days after surgery. Penicillium genus could secrete penicillin G and griseofulvin, which have antibacterial and antifungal effects respectively. These compounds may also inhibit the abnormal growth of some bacteria and fungi in the intestinal tract, thus maintaining intestinal microbiota homeostasis [22, 38]. A decrease of the relative abundance of Penicillium may further worsen intestinal microbiota dysbiosis in SBS rats. GLP-2 treatment could partially reverse the decrease of the relative abundance of Penicillium in SBS rats, which may have beneficial effects on the balance of intestinal microbiota. Coexisting in the gut, intestinal fungi and bacteria interact directly or indirectly with each other [11, 39]. A growing number of studies confirmed that the bacterial-fungal interactions played an important role in the occurrence and exacerbation of many diseases, such as IBD, primary sclerosing cholangitis, neurological disease, and even cancer [8–10, 20, 40]. We herein investigated the bacterial-fungal correlation patterns in SBS rats and found disrupted bacteria–fungi correlations existed in the colonic content of SBS rats. Fortunately, GLP-2 treatment might partially re-build the interkingdom interaction in SBS rats.

However, our study also has some limitations, such as small sampling size and lack of sampling over time. Our team’s previous published results confirmed that the 16S sequencing data were reliable under consistent sampling process and the difference was small within the group but significant between groups [2, 7]. From the Anosim consistency analysis in this study, we found that the intra group differences of our samples were small, but the inter group differences were large. Therefore, we still found a lot of bacterial and fungal taxa with significant differences in case of existing small sample size. This also indirectly indicated that the intestinal flora of SBS rats was in severe dysbiosis and GLP-2 might attenuate the intestinal flora dysbiosis caused by SBS to a certain extent. We set the sampling time at the 22nd day after operation, because at this time point the intestinal adaptation of SBS rats had reached the peak while the residual intestinal internal environment and the intestinal flora of SBS rats has reached a relatively stable state so that the results would be more reliable. However, if we could enrich the sampling time point to declare the effects of whole process of intestinal adaptation and GLP-2 treatment on intestinal flora of SBS, this study would be more meaningful.

## Conclusions

**Fig. 11:**
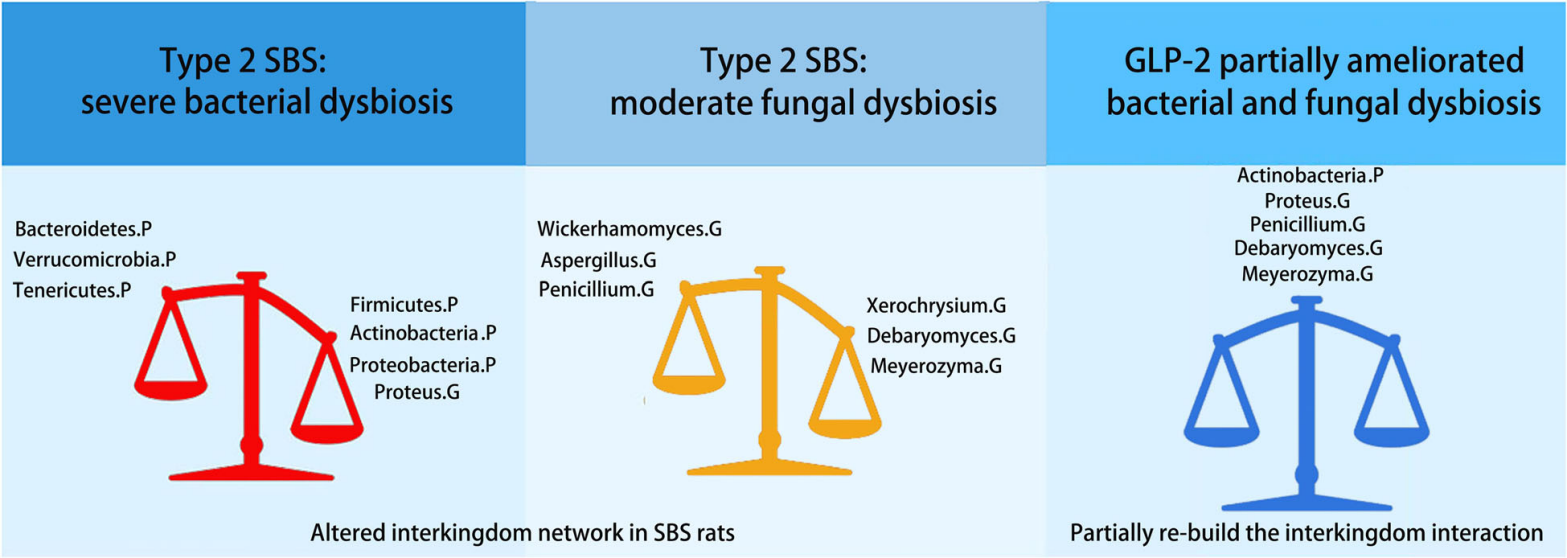
Graphic abstract. There were severe bacterial dysbiosis and moderate fungal dysbiosis in the colon of type 2 SBS rats, while GLP-2 might have potential therapeutic effect of these dysbiosis

We found that severe colonic bacterial dysbiosis of SBS rats was accompanied by significant fungal dysbiosis, and GLP-2 treatment might partially ameliorate the intestinal bacterial and fungal dysbiosis in SBS rats (Fig. 11).

## Supplementary Information

**Additional file 1: Figure S1.** Anosim similarity analysis of intestinal bacteria. [A], [C] and [E] Anosim similarity analysis based on weighted_unifrac distance rarefaction curves of each sample. [B], [D] and [F] Anosim similarity analysis based on Bray_curtis_OTU distance rarefaction curves of each sample.

**Additional file 2: Figure S2.** Anosim similarity analysis of intestinal fungi. [A], [C] and [E] Anosim similarity analysis based on weighted_unifrac distance rarefaction curves of each sample. [B], [D] and [F] Anosim similarity analysis based on Bray_curtis_OTU distance rarefaction curves of each sample.

**Additional file 3:**

## Data Availability

The sequencing data for our study are available in the NCBI Sequence Read Archive under accession no. PRJNA677835 (https://www.ncbi.nlm.nih.gov/sra/PRJNA677835) and PRJNA677844 (www.ncbi.nlm.nih.gov/sra/PRJNA677844) after the indicated release date.

## Abbreviations

SBS: Short bowel syndrome
GLP-2: Glucagon-like peptide 2
ITS: Internal transcribed spacer
IBD: Inflammatory bowel disease
EEC: Enteric endocrine cells
PCoA: Principal coordinate analysis
PLS-DA: Partial least-squares discrimination analysis
SCFAs: Short-chain fatty acids

## References

[1] Cheng W, Wang K, Zhao Z, Mao Q, Wang G, Li Q, Fu Z, Jiang Z, Wang J, Li J (2020). Exosomes-mediated transfer of miR-125a/b in cell-to-cell communication: a novel mechanism of genetic exchange in the intestinal microenvironment. Theranostics.

[2] Huang Y, Chen A, Guo F, Wang J, Li Y (2020). Severe intestinal Dysbiosis in rat models of short bowel syndrome with Ileocecal resection. Dig Dis Sci.

[3] Piper HG (2018). Intestinal microbiota in short bowel syndrome. Semin Pediatr Surg.

[4] Piper HG, Fan D, Coughlin LA, Ho EX, McDaniel MM, Channabasappa N, Kim J, Kim M, Zhan X, Xie Y (2017). Severe gut microbiota Dysbiosis is associated with poor growth in patients with short bowel syndrome. JPEN J Parenter Enteral Nutr.

[5] Engstrand Lilja H, Wefer H, Nyström N, Finkel Y, Engstrand L (2015). Intestinal dysbiosis in children with short bowel syndrome is associated with impaired outcome. Microbiome.

[6] Goulet O, Joly F (2010). Intestinal microbiota in short bowel syndrome. Gastroenterologie clinique et biologique.

[7] Huang Y, Guo F, Li Y, Wang J, Li J (2017). Fecal microbiota signatures of adult patients with different types of short bowel syndrome. J Gastroenterol Hepatol.

[8] Lemoinne S, Kemgang A, Ben Belkacem K, Straube M, Jegou S, Corpechot C, Chazouillères O, Housset C, Sokol H (2020). Fungi participate in the dysbiosis of gut microbiota in patients with primary sclerosing cholangitis. Gut.

[9] Sokol H, Leducq V, Aschard H, Pham HP, Jegou S, Landman C, Cohen D, Liguori G, Bourrier A, Nion-Larmurier I, Cosnes J, Seksik P, Langella P, Skurnik D, Richard ML, Beaugerie L (2017). Fungal microbiota dysbiosis in IBD. Gut.

[10] Richard ML, Liguori G, Lamas B, Brandi G, da Costa G, Hoffmann TW, Pierluigi Di Simone M, Calabrese C, Poggioli G, Langella P (2018). Mucosa-associated microbiota dysbiosis in colitis associated cancer. Gut Microbes.

[11] Jun X, Ning C, Yang S, Zhe W, Na W, Yifan Z, Xinhua R, Yulan L (2020). Alteration of fungal microbiota after 5-ASA treatment in UC patients. Inflamm Bowel Dis.

[12] Gribble FM, Reimann F (2019). Function and mechanisms of enteroendocrine cells and gut hormones in metabolism. Nat Rev Endocrinol.

[13] Wu J, Ren W, Li L, Luo M, Xu K, Shen J, Wang J, Chang G, Lu Y, Qi Y, Xu B, He Y, Hu Y (2018). Effect of aging and glucagon-like peptide 2 on intestinal microbiota in SD rats. Aging Dis.

[14] Magoč T, Salzberg SL (2011). FLASH: fast length adjustment of short reads to improve genome assemblies. Bioinformatics (Oxford, England).

[15] Edgar RC (2013). UPARSE: highly accurate OTU sequences from microbial amplicon reads. Nat Methods.

[16] Edgar RC, Haas BJ, Clemente JC, Quince C, Knight R (2011). UCHIME improves sensitivity and speed of chimera detection. Bioinformatics (Oxford, England).

[17] Caporaso JG, Kuczynski J, Stombaugh J, Bittinger K, Bushman FD, Costello EK, Fierer N, Peña AG, Goodrich JK, Gordon JI, Huttley GA, Kelley ST, Knights D, Koenig JE, Ley RE, Lozupone CA, McDonald D, Muegge BD, Pirrung M, Reeder J, Sevinsky JR, Turnbaugh PJ, Walters WA, Widmann J, Yatsunenko T, Zaneveld J, Knight R (2010). QIIME allows analysis of high-throughput community sequencing data. Nat Methods.

[18] Richard ML, Sokol H (2019). The gut mycobiota: insights into analysis, environmental interactions and role in gastrointestinal diseases. Nat Rev Gastroenterol Hepatol.

[19] Lang S, Duan Y, Liu J, Torralba MG, Kuelbs C, Ventura-Cots M, Abraldes JG, Bosques-Padilla F, Verna EC, Brown RS, Vargas V, Altamirano J, Caballería J, Shawcross D, Lucey MR, Louvet A, Mathurin P, Garcia-Tsao G, Ho SB, Tu XM, Bataller R, Stärkel P, Fouts DE, Schnabl B (2020). Intestinal fungal Dysbiosis and systemic immune response to Fungi in patients with alcoholic hepatitis. Hepatology.

[20] Forbes JD, Bernstein CN, Tremlett H, Van Domselaar G, Knox NC (2018). A fungal world: could the gut Mycobiome be involved in neurological disease?. Front Microbiol.

[21] Jiang TT, Shao TY, Ang WXG, Kinder JM, Turner LH, Pham G, Whitt J, Alenghat T, Way SS (2017). Commensal fungi recapitulate the protective benefits of intestinal bacteria. Cell Host Microbe.

[22] Chin VK, Yong VC, Chong PP, Amin Nordin S, Basir R, Abdullah M (2020). Mycobiome in the gut: a multiperspective review. Mediat Inflamm.

[23] Lapthorne S, Pereira-Fantini PM, Fouhy F, Wilson G, Thomas SL, Dellios NL, Scurr M, O'Sullivan O, Ross RP, Stanton C (2013). Gut microbial diversity is reduced and is associated with colonic inflammation in a piglet model of short bowel syndrome. Gut Microbes.

[24] Levesque CL, Turner J, Li J, Wizzard P, St Pierre B, Lim D, Wales P (2017). In a neonatal piglet model of intestinal failure, Administration of Antibiotics and Lack of enteral nutrition have a greater impact on intestinal microflora than surgical resection alone. JPEN J Parenter Enteral Nutr.

[25] Davidovics ZH, Carter BA, Luna RA, Hollister EB, Shulman RJ, Versalovic J (2016). The fecal microbiome in pediatric patients with short bowel syndrome. JPEN J Parenter Enteral Nutr.

[26] Engelstad HJ, Barron L, Moen J, Wylie TN, Wylie K, Rubin DC, Davidson N, Cade WT, Warner BB, Warner BW (2018). Remnant small bowel length in pediatric short bowel syndrome and the correlation with intestinal Dysbiosis and linear growth. J Am Coll Surg.

[27] Shin NR, Whon TW, Bae JW (2015). Proteobacteria: microbial signature of dysbiosis in gut microbiota. Trends Biotechnol.

[28] Schönfeld P, Wojtczak L (2016). Short- and medium-chain fatty acids in energy metabolism: the cellular perspective. J Lipid Res.

[29] Qing Y, Xie H, Su C, Wang Y, Yu Q, Pang Q, Cui F (2019). Gut microbiome, short-chain fatty acids, and mucosa injury in young adults with human immunodeficiency virus infection. Dig Dis Sci.

[30] Ma Y, Hu C, Yan W, Jiang H, Liu G (2020). Lactobacillus pentosus increases the abundance of Akkermansia and affects the serum Metabolome to alleviate DSS-induced colitis in a murine model. Front Cell Dev Biol.

[31] Wang L, Tang L, Feng Y, Zhao S, Han M, Zhang C, Yuan G, Zhu J, Cao S, Wu Q, Li L, Zhang Z (2020). A purified membrane protein from Akkermansia muciniphila or the pasteurised bacterium blunts colitis associated tumourigenesis by modulation of CD8(+)T cells in mice. Gut.

[32] Depommier C, Everard A, Druart C, Plovier H, Van Hul M, Vieira-Silva S, Falony G, Raes J, Maiter D, Delzenne NM (2019). Supplementation with Akkermansia muciniphila in overweight and obese human volunteers: a proof-of-concept exploratory study. Nat Med.

[33] Xu Y, Wang N, Tan HY, Li S, Zhang C, Feng Y (2020). Function of Akkermansia muciniphila in obesity: interactions with lipid metabolism, immune response and gut systems. Front Microbiol.

[34] Morgan XC, Tickle TL, Sokol H, Gevers D, Devaney KL, Ward DV, Reyes JA, Shah SA, LeLeiko N, Snapper SB, Bousvaros A, Korzenik J, Sands BE, Xavier RJ, Huttenhower C (2012). Dysfunction of the intestinal microbiome in inflammatory bowel disease and treatment. Genome Biol.

[35] Fifi AC, Bayes L, Ehrenpreis ED, Chavez H (2020). Prevalence of bloodstream infections in children with short-bowel syndrome with a central line presenting to emergency department with fever. JPEN J Parenter Enteral Nutr.

[36] Raphael BP, Fournier G, McLaughlin SR, Puder M, Jones S, Flett KB (2020). Antibiotic susceptibility and therapy in central line infections in pediatric home parenteral nutrition patients. J Pediatr Gastroenterol Nutr.

[37] El Mouzan M, Wang F, Al Mofarreh M, Menon R, Al Barrag A, Korolev KS, Al Sarkhy A, Al Asmi M, Hamed Y, Saeed A (2017). Fungal microbiota profile in newly diagnosed treatment-naïve children with Crohn's disease. J Crohn’s Colitis.

[38] Banani H, Marcet-Houben M, Ballester AR, Abbruscato P, González-Candelas L, Gabaldón T, Spadaro D (2016). Genome sequencing and secondary metabolism of the postharvest pathogen Penicillium griseofulvum. BMC Genomics.

[39] Peleg AY, Hogan DA, Mylonakis E (2010). Medically important bacterial-fungal interactions. Nat Rev Microbiol.

[40] Imai T, Inoue R, Kawada Y, Morita Y, Inatomi O, Nishida A, Bamba S, Kawahara M, Andoh A (2019). Characterization of fungal dysbiosis in Japanese patients with inflammatory bowel disease. J Gastroenterol.

