# Supplementary figures and images for: Therapeutic potential of an intestinotrophic hormone, glucagon-like peptide 2, for treatment of type 2 short bowel syndrome rats with intestinal bacterial and fungal dysbiosis
Source: BMC Infect Dis. 2021 Jun 16;21:583. doi: 10.1186/s12879-021-06270-w (PMC8207711; doi:10.1186/s12879-021-06270-w)

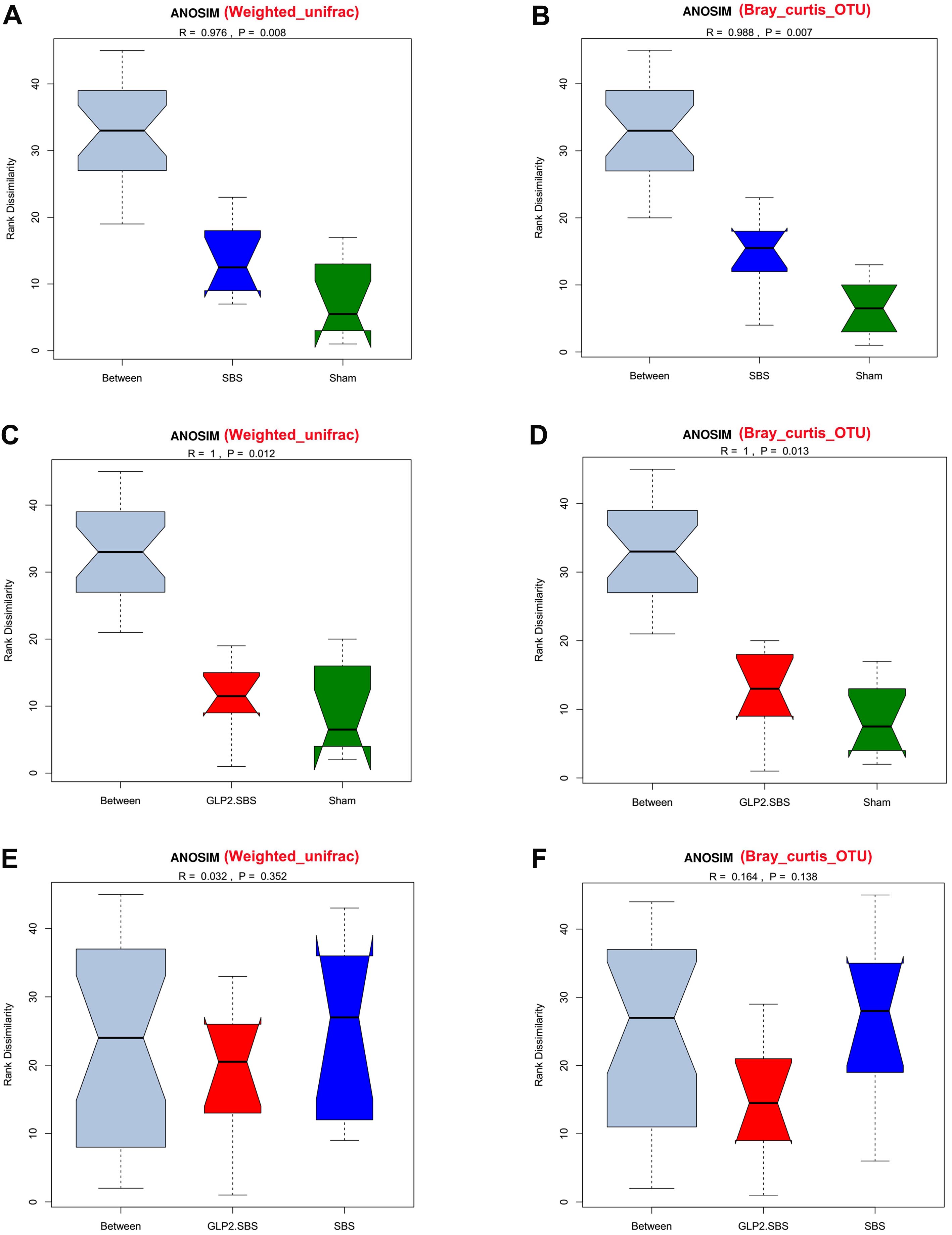

Supplement: Supplementary file 1 — Additional file 1: Figure S1. Anosim similarity analysis of intestinal bacteria. [A], [C] and [E] Anosim similarity analysis based on weighted_unifrac distance rarefaction curves of each sample. [B], [D] and [F] Anosim similarity analysis based on Bray_curtis_OTU distance rarefaction curves of each sample. [file 12879_2021_6270_MOESM1_ESM.jpg]

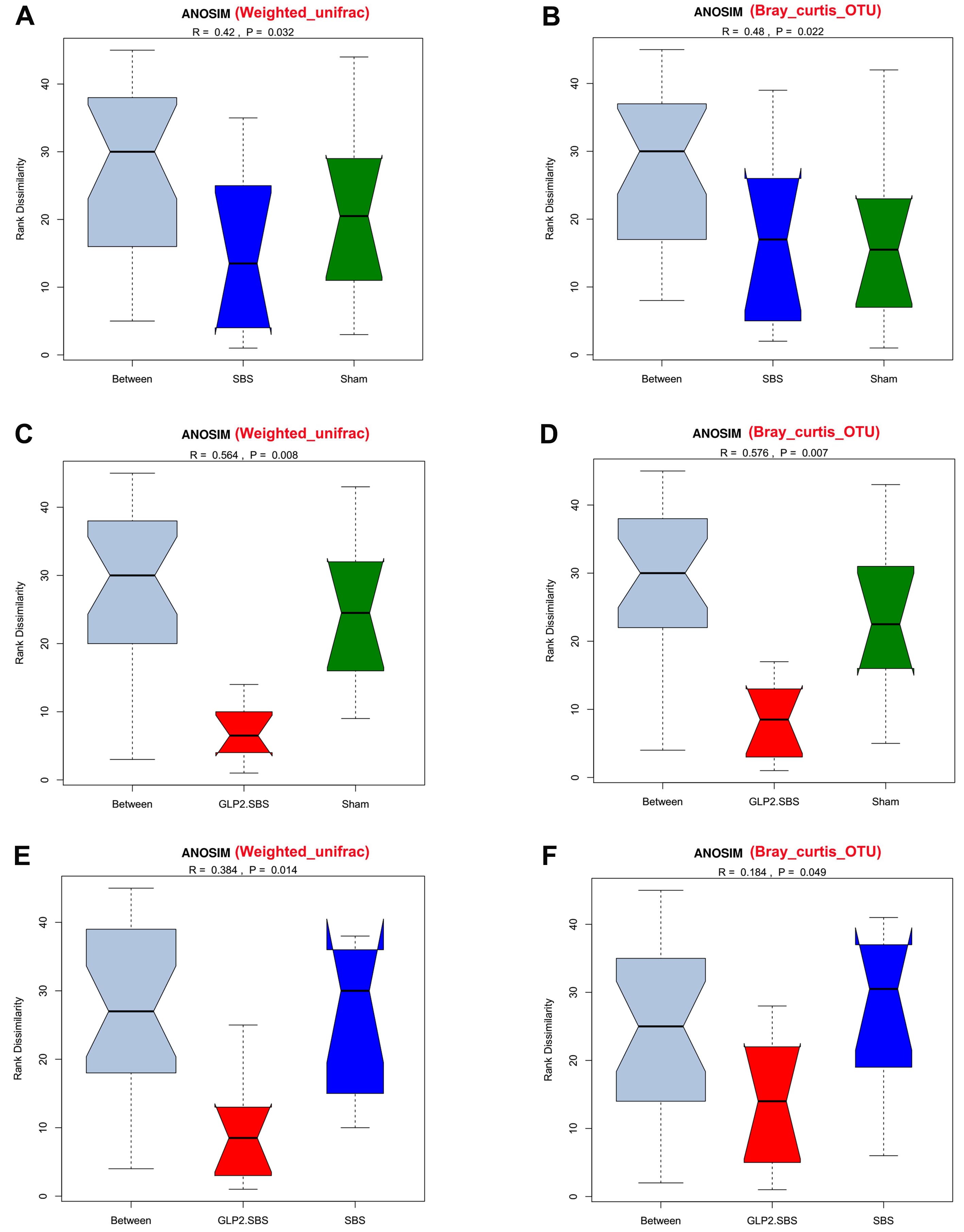

Supplement: Supplementary file 2 — Additional file 2: Figure S2. Anosim similarity analysis of intestinal fungi. [A], [C] and [E] Anosim similarity analysis based on weighted_unifrac distance rarefaction curves of each sample. [B], [D] and [F] Anosim similarity analysis based on Bray_curtis_OTU distance rarefaction curves of each sample. [file 12879_2021_6270_MOESM2_ESM.jpg]
