# Supplementary material for: Therapeutic potential of an intestinotrophic hormone, glucagon-like peptide 2, for treatment of type 2 short bowel syndrome rats with intestinal bacterial and fungal dysbiosis
Source: BMC Infect Dis. 2021 Jun 16;21:583. doi: 10.1186/s12879-021-06270-w (PMC8207711; doi:10.1186/s12879-021-06270-w)
Supplement: Supplementary file 3 — Additional file 3: [file 12879_2021_6270_MOESM3_ESM.docx]

REVISION 1 REPORTS

### Reports

**Amanda B. Blake**21-FEB-21

**FEEDBACK FOR AUTHOR(S)**

The manuscript entitled “Therapeutic potential of an intestinotrophic hormone, glucagon-like peptide 2, for treatment of type2 short bowel syndrome rats with intestinal bacterial and fungal dysbiosis” is answering an important research question and is very interesting. I do believe that the results found by the authors can contribute to our understanding of SBS and bacterial/fungal dysbiosis. However, some of the conclusions presented are misleading or overstated and are presented in a way that is unclear. I really would encourage the authors to clearly discuss the limitations of the study and to simplify the presentation of results so as not to mislead the reader.

The statistical analysis needs some clarification/revision. PCoA plots give graphical representation of bacterial/fungal communities but cannot alone be used to determine significant differences in clustering. I suggest adding an ANOSIM test, or be clear that the apparent difference in clustering is speculation. Also, in line 138-139 is it meant to say that taxa were compared with this R package and not species? Was it used to compare the alpha diversity measures too (not specified)? None of the samples are paired though, so Wilcoxon testing (based on two paired groups) should not be used. And if Kruskal Wallis testing was used, then it would require a post hoc test to show differences between the 3 groups. Please specify which post hoc test was used and correct for multiple comparisons. Additionally, because many taxa are being compared, a correction for false discovery rate is needed. Additionally, the statistics used in GraphPad Prism are not clear. Which tests were used? Parametric testing should not be used as the sample size is too low in each group to adequately meet assumptions of the parametric test. Therefore, only Kruskal Wallis testing should be used with post hoc test for multiple comparisons and FDR adjustment. The way it is currently worded, it is not clear which nonparametric testing was used or if it included any corrections/adjustments of the p-value. Furthermore, nonparametric data should be reported with median and range, not mean and standard deviation. Please change these in all tables and figures.

Additionally, care needs to be taken when discussing the changes seen in Clostridium. Species within the Clostridium genus are not always beneficial or anti-inflammatory and generalizing them to be so can be misleading. There are numerous species included in the Clostridium genus that are not beneficial, including C. difficile and C. perfringens. Clostridium spp. can also be increased in patients with gastrointestinal disease compared to healthy patients (https://doi.org/10.1186/gb-2012-13-9-r79).

Finally, while the study was designed well to answer the research question, it does have some limitations that should be discussed, possibly in a separate paragraph at the end of the discussion section. These limitations include the small sampling size and lack of sampling over time.

Please see the following for specific comments:

• Line 24: inconsistent spelling of “type2/ type 2” SBS; fix throughout

• Line 28-29: “While data are restricted…” sentence is unclear. Would suggest changing to “There are limited studies on intestinal fungal dysbiosis.”

• Line 29: The term “disorder” and “dysbiosis” seem to be used interchangeably throughout the manuscript. For consistency and accuracy, I suggest using “dysbiosis” instead.

• Line 35: The acronym used for the SBS placebo group (SBS) is very confusing because when talking about SBS rats, it is unclear if you mean both SBS groups (treated and untreated) or only the placebo group. I strongly suggest changing the acronym of the placebo group to pSBS, SBS+p, or SBS+placebo.

• Line 40: “…Proteobacteria increased significantly in SBS rats,…” –does this mean in both groups of SBS rats combined compared to sham?

• Line 41-42: delete sentence “ GLP-2 treatment could partially ameliorate…”. Could add that GLP-2 treatment decreased Proteobacteria and increased Clostrium relative to the SBS+placebo rats.

• Line 42-43: delete “Meanwhile,…” sentence

• Line 45-46: revise sentence “ GLP-2 could partially…” to describe the results and not draw conclusions from them (i.e., which fungal groups were altered by GLp-2 treatment compared to placebo?)

• Line 47: delete one “interkingdom”

• Line 54: If the term is only used once, it does not need an abbreviation (IF)

• Line 58: insert “or between “liver injury” and “catheter related infection”

• Line 76: replace “exhibited” with “is”

• Line 86-87: “GLP-2 is the most crucial factor in current intestinal rehabilitation therapy of SBS.”—Is GLP-2 currently being used to treat SBS, in humans or other species? What makes it a crucial factor in rehabilitation therapy, more so than, for example, limiting carbohydrate consumption or controlling bacterial overgrowth?

• Line 106: Delete “a” in sentence, “…and were given a saline as placebo.”

• Line 110: Where is the company Creative Peptides based out of and in what volume were the treatment administered? This information should be included.

• Line 113: How were the rats sacrificed, as some methods may affect the intestinal microbiota

• Line 115-116: Please include the full method of how the stool DNA was extracted. The two references only state that DNA was extracted according to the manufacturer’s instructions with minor modifications. However, those minor modifications are necessary for this study to be reproducible.

• Line 119: “fungal” is misspelled

• Line 130: should provide citation for Greengenes, R packages and LEfSe to give credit to the people who created these

• Line 149: what is the mean and standard deviation of 16S tags and was there a rarefied sequencing depth?

• Line 151: revise section header

• Line 162: are there p-values for the statistically significant differences? Is this sentence describing both SBS groups?

• Line 164: Please see comments on statistical analysis, but PCoA alone cannot declare groups different. ANOSIM can.

• Line 194: “…manifested as significant inhibition…”

• Line 223: showed

• Line 232-233: combine the two sentences to: “Figure 8 showed profiling of each sample at the class level and gave an idea of the proportion of different classes in each sample.” It is not species profiling because the analysis did not go down to the species taxonomic level, so please delete the word “species”.

• Line 233: Furthermore,…

• Line 234: again, the analysis did not go to species level. Were all of the genus levels in the 5 most abundant classes analyzed? It seems like only two classes were analyzed to genus level in table 3.

• Line 244: add “no significant difference between the three groups” to the end of this sentence and delete the sentence “ While GLP-2 treatment…”

• Line 263: Furthermore

• Line 296: intestinal bacterial dysbiosis

• Line 302-303: …environment of intestinal microbiota, thus leading to…

• Line 307 and 309: could replace “structures” with “communities”

• Line 323: when discussing other studies’ results, should be in present tense instead of past tense. i.e., LPS results in… and further leads to… SCFA have many

• Line 331: epithelial cells

• Line 333: “in line with previous research…”

• Line 343: “However, previous studies…”

• Line 348: ”…and increased the relative abundance of Clostridium…”

• Line 349-351: delete sentence beginning with “Therefore…”

• Line 352-353: “…the colonic fungi of SBS rats was abnormal, indicated by increased OTU…”

• Line 373: Furthermore

• Line 389: “…SBS group exhibited significant changes…”

• Line 391: Be cautious using the term “reverse” or “inhibit” to describe the changes seen in the SBS+GLP-2 groups compared to the SBS+placebo group. This study did not include sampling at multiple time points and therefore it is a bit overreaching to state that the treatment reversed or inhibited changes because it implies directionality. Could simplify much of the results by simply stating the changes seen.

• Line 393: delete sentence “It is sufficient to indicate the therapeutic effect of GLP-2.”

• Line 393-397: “…secrete penicillin G and griseofulvin, which have antibacterial and antifungal effects, respectively. These compounds may also inhibit the abnormal growth of some bacteria and fungi…”

• Figure 1C: It is difficult to tell the difference between colors on this graph. Could one line be plotted for each group with mean and standard deviation shown?

• Figure legends: be consistent with the bracketing of subsections within the figures. i.e., if using square brackets around [A] or [B], be consistent throughout the figure legend.

**FEEDBACK FOR EDITOR**

Your recommendation

Revise

Is the study design appropriate to answer the research question (including the use of appropriate controls), and are the conclusions supported by the evidence presented?

No, but these points can be addressed with revisions

Comments: The authors use appropriate controls and they are answering an important research question. However, the conclusions are severely overstated and the limitations of the study are not described.

It is misleading to claim that the GLP-2 treatment ameliorated the bacterial/fungal dysbiosis when so few taxa were altered and they still mostly resembled the placebo treated rats. For example, in Line 170: it is claimed that the bacterial phyla differed dramatically between the three groups. However, only one bacterial phyla was different between the SBS+GLP-2 and SBS+placebo groups, with those two groups appearing largely similar. Additionally, on Line 349 it is claimed that Clostridium genus is anti-inflammatory and since treatment with GLP-2 appeared to increase its abundance, that treatment is beneficial. This is a massive overstatement of conclusions because species in the clostridium genus are not always beneficial (e.g., C. difficile, C. perfringens, etc.) and Clostridium abundance is also increased in patients with gastrointestinal disease compared to healthy patients. Additionally, the results presented in table 2 show that Clostridium spp. increased in SBS+placebo rats compared to sham as well, and GLP-2 treatment further increased Clostridium spp. perhaps worsening the dysbiosis.

Furthermore, the very small sample size is a large limitation of the study that should be discussed, along with any other limitations of the study.

Therefore, I suggest heavy revision (including results section headings, abstract, etc.) on conclusions drawn from the evidence presented, limited to ‘GLP-2 treatment altered bacterial and fungal taxa in SBS rats’ so as not to be misleading to the reader.

Are the methods sufficiently described to allow the study to be repeated?

No, but these points can be addressed with revisions

Comments: Line 110: specify where the company “Creative Peptides” is located and which GLP-2 was used (rat?). Also, what volume was it administered in.

Line 113: What is the method used to sacrifice the rats as this can sometimes affect the microbiota.

Line 116: both citations say they extracted DNA following “manufacturer’s instructions with minor modifications” but neither states what the minor modifications are. This information is essential to being able to replicate this study and should be included in this manuscript.

Is the use of statistics and treatment of uncertainties appropriate?

No

Comments: I am not familiar with correlation networks and therefore cannot judge if this part of the analysis was done properly. However, the rest of the analysis needs major revision.

Line 135: PCoA plots give graphical representation of bacterial/fungal communities but cannot alone be used to determine significant differences in clustering. The authors need to add an ANOSIM test, or be clear that the apparent difference in clustering is speculation.

Line 138-139: The authors compared a lot more than species. Do they mean to say they compared all taxa with this R package? Did they use it to compare the alpha diversity measures too (not specified)? None of the samples are paired though, so Wilcoxon testing (based on two paired groups) should not be used. And if Kruskal Wallis testing was used, then it would require a post hoc test to show differences between the 3 groups. The authors need to specify which post hoc test was used and correct for multiple comparisons. Additionally, because they are comparing so many taxa, a correction for false discovery rate is needed.

Line 145: The statistics used in GraphPad Prism are not clear. The authors do not name the tests they used. They should not use parametric testing as the sample size is too low in each group to adequately meet assumptions of the parametric test. Therefore, they should only be using Kruskal Wallis testing with post hoc test for multiple comparisons and FDR adjustment. The way it is currently worded, it is not clear which nonparametric testing they used or if it included any corrections/adjustments of the p-value.

Is the presentation of the work clear?

No, it needs some language corrections before being published

Comments: While overall, the flow of the manuscript is well written, there are over 30 minor spelling/grammatical errors. Additionally, use of terminology and spelling needs to be more consistent. For example, the authors frequently use the term “disorder” throughout the manuscript, when “dysbiosis” may be a more accurate term. The term “reverse” is also used frequently to describe differences between the SBS+GLP-2 group and SBS+placebo group of rats. This term may be misleading since the study did not look at the effect of GLP-2 treatment over time. So, to be clear, conclusions cannot be drawn whether treatment was reversing or preventing the changes seen in the SBS+placebo rats. Either a more neutral term should be used, or an explanation of this limitation should be included in the discussion. Finally, and perhaps most importantly, it is not clear when the SBS rats are discussed, if this means both SBS groups or only the SBS+placebo group. For the purpose of clarity, the abbreviation of the group of rats that received placebo and SBS should be changed throughout the manuscript and figures (i.e., pSBS, SBS+placebo, SBS+p, etc.). If the SBS groups were combined for some analysis, this needs to be made clear in the statistical analysis section.

Are the images in this manuscript (including electrophoretic gels and blots) free from apparent manipulation?

Yes

Confidential Comments to the Editor

**Andreas Vegge**30-DEC-20

**FEEDBACK FOR AUTHOR(S)**

The study by Hu et al, describes microbial changes following a type 2 resection in a rat model and investigate changes in microbial and fungal composition both from SBS and also from GLP-2 treatment in SBS treated animals. It is a challenging animal model and the authors should be congratulated trying contribute with new knowledge in a condition as difficult at SBS.

The paper reads well, although I feel data are perhaps overinterpreted a few times. Below is a number of concrete suggestions. However, my primary concern with the study is the following:

It is good that you include both sham, SBS and SBS+GLP-2. However a key problem with the study is if the colonic samples really are taking from comparable locations? e.g. 3 cm proximal or distal to resection site, in one group you would get a small intestinal sample and in the SBS a samples from the distal colon. It is really more the effect of SBS or sampling site being studies and not the effect of GLP-2. It would have been preferable to also take the colonic samples at the time of resection so you get pre and post adaption to resection. Another key challenge is the very low n. I would say that higher n would be preferable in all groups, there is especially a very large variation on some parameters in the SBS+GLP-2 group. Alternatively a 2x2 design could have been applied, where you also included a SHAM+GLP-2 group.

Abstract:

consider rewording line 27-28, e.g. previous studies/findings, rather than refering to a specific study

Sentence line 28. “while data are restricted in the intestinal fungal disorder” this is unclear to me, do you mean limited data are available for intestinal fungal disorder?

Line 30 – please use plural language.

Methods:

The study would benefit by some information on enteral diet. In many studies with SBS models a certain degree of parenteral nutrition is needed. I think more info needs to be added on postsurgical recovery and diet used.

At line 113 you state that the content samples were obtained 3 cm from the anastomotic site. Was samples collected proximal or distal to the anastomosis? In either case you are not really sampling from the same location in the sham and SBS animals. Figure 1C would indicate that decrease in OUT richness is more driven by SBS or actually anatomical sampling site than anything else. In line 154, you state that it is SBS that induced this, but are we not just looking at a colonic sample from two very different anatomical sites? Can you please elaborate on this also in discussion?

Results

Figure 1C and 1D are more or less showing the same with 1D showing OUT at saturation, you could only show one of these.

Overall the OTU data and the diversity indices indicate very similar data for SBS and SBS+GLP-2, why do we not see an effect of GLP-2 in these?

Line 161-163, it appears the UniFrac distance is different from SHAM and both SBS groups, but no difference between SBS groups?

The PCoA analysis is really nice, I would also suggest to add to the text the how much of the variability is captured by PC1 and PC2.

Line 170, you state fecal flora, but in methods it appears you “only” collected colonic samples. Please clarify.

The findings in figure 3G-L requires further explanation either in results of discussion. Essentially you cannot quantify or detect these phylum, and I am questioning whether we are really comparing the sampling site when we compare across SBS and non SBS groups. i.e. what is a consequence of resection induces changes vs just changes in composition depending on anatomical site.

There appears to be a very large variation in Chao and ACE in the SBS+GLP-2 group, which could indicate an underpowered study. Secondly it is peculiar whey larger variation is observed in SBS+GLP-2 vs SBS alone, do you have any speculations on this.

LIne 220-222. I am not really sure you based on the ata presented in Fig 6 D-E can say that GLP-2 treat partially reversed overgrowth. I follow that it couples with figure 6C, but really you have a very large variation in the SBS+GLP-2 group, so I think you need to soften up this statement.

Discussion

LIne 322, it is difficult to understand what data on Proteous is you own and what are from other studies. There is no data in the present paper to demonstrate the Proteus induces LPS and upregulation of pro-inflammatory cytokines, please be very clear that this was observed in other studies.

I think should include more discussion on Akkermansia and some of the findings for this related to the systemic immune system, specifically the work on Akkermansia Mucinophila.

Line 342 – re-phrase, just “However, effective therapies for intestinal…”

LIne 350, very strong statements about the potential benefit of GLP-2 based on the present data. I would recommend to be a bit more cautious not overinterpreting.

Line 353-358 – please rewrite discussion on colonic fungi, you do not that the Chao or Ace are change from sham by SBS+GLP-2. However you also do not observe that SBS+GLP-2 is different from SBS. It is very hard to make any conclusions with this low number of samples and the considerable variation observed in SBS+GLP-2.

Minor editorial comments:

Please correct type2 to type 2. Explain type 2 early on the introduction, please elaborate on this in line 69.

Would advice that you adjust the figure legends so same terminology is used both in text and in figures for the groups e.g. SBS+GLP-2, which is named different in for instance figure 6 and figure 10.

**FEEDBACK FOR EDITOR**

Your recommendation

Revise

Is the study design appropriate to answer the research question (including the use of appropriate controls), and are the conclusions supported by the evidence presented?

No, but these points can be addressed with revisions

Comments: It is good that they have both sham, SBS and SBS+GLP-2. However a key problem with the study is if the colonic slamples really are taking from comparable locations? e.g. 3 cm proximal or distal to resection site, in one group you would get a small intestinal sample and in the SBS a samples from the distal colon. It is really more the effect of SBS or sampling site being studies and not the effect of GLP-2. It would have been preferable to also take the colonic samples at the time of resection so you get pre and post adaption to resection. Another key challenge is the very low n. I would say that higher n is needed in all groups, there is especially a very large variation on some parameters in the SBS+GLP-2 group.

Are the methods sufficiently described to allow the study to be repeated?

Yes

Is the use of statistics and treatment of uncertainties appropriate?

No

Comments: Most of the statistics are okay but more info is lacking. They state adjusted p-value, but not which correction is made. I also struggle that they use parametric statics for low n. No information given if the show mean, median, SD. The PCoA and associated are really nice.

Is the presentation of the work clear?

Yes

Are the images in this manuscript (including electrophoretic gels and blots) free from apparent manipulation?

Not applicable

Confidential Comments to the Editor

Overall it is an interesting study. However, my main concerns are really related to the fact that they perform the study in very low n for all groups and they have a high degree of variation. They should also have done a sample at the time of surgery so they could compare within animal and get pre and post intestinal adaption. It is unclear where they sample the colonic material and if the sampling site is really comparable among groups.

The study is very descriptive and not really have a concrete hypothesis for a given change. Also the findings is really limited to "only" 16s and ITS, where I am also lacking a link to let say the intestinal adaptation per se. I think 10 figures is quite a lot for the data presented, where a large part could be summarized in tables. That said the figure work is really nice and explains the findings well.

Study requires statistical review given the low n.

REVISION 2 REPORTS

Reports

**Andreas Vegge**22-APR-21

**FEEDBACK FOR AUTHOR(S)**

The authors have done a good job in answering the points raised by the reviewers. My key concern is really relating to sample size, and I think it is prudent and good that you now include statements also highlighting the limitation of the study due to the sample size and have soften up on conclusions. I realize these are difficult models to work with.

My points have been clarified and manuscript has been adjusted. I do not have further comments.

**FEEDBACK FOR EDITOR**

Your recommendation

Accept

Is the study design appropriate to answer the research question (including the use of appropriate controls), and are the conclusions supported by the evidence presented?

Yes

Are the methods sufficiently described to allow the study to be repeated?

Yes

Is the use of statistics and treatment of uncertainties appropriate?

Yes

Is the presentation of the work clear?

Yes

Are the images in this manuscript (including electrophoretic gels and blots) free from apparent manipulation?

Yes

Confidential Comments to the Editor

I think the authors have done a nice job clarifying concerns and questions I had. One key limitation of the study is the quite small sample size, however, difficult for the authors to work around this without having to do additional in vivo experiments. We should keep in mind that these are challenging models to work with. I think the authors have softened up on key statements, to also acknowledge the limitations of the study.

I have no further comments.

**Amanda B. Blake**21-APR-21

**FEEDBACK FOR AUTHOR(S)**

The revised manuscript is much improved and I commend the authors for the great work that went into addressing comments. It is excellent that they were able to obtain statistical advice from a statistics expert. Very well done!

In response to changing the acronym for SBS saline group, thank you for the explanation of the difficulties involved in changing it. I understand, and the acronym does not need to be changed. However, this does not change my original comment that the use of “SBS rats” is very confusing, when this term could be describing both groups (because they both had SBS procedure) or only the saline group. Even though the term “SBS group” is clearly stated in the abstract, “SBS rats” is easily misconstrued to mean the same thing. There are still instances where “SBS rats” is used to describe results that apply to both the SBS saline and GLP2.SBS groups. For example, line 173: “compared with Sham group, the α diversity of intestinal bacteria in SBS rats was significantly decreased…” The alpha diversity was also decreased in GLP2.SBS rats compared to Sham, and this is not mentioned elsewhere in this paragraph. Instead, saying “compared with Sham group, the α diversity of intestinal bacteria in SBS and GLP2.SBS rats was significantly decreased…” makes it clearer that these results apply to both groups. Please make the additional changes for increased clarity:

Line 202: “…in SBS and GLP2.SBS rats, indicating that the intestinal bacterial flora of rats that underwent SBS procedure has been seriously disturbed (Figure 3G-3L).”

Line 205: “SBS, regardless of GLP2 administration, resulted in an obvious reduction of multiple bacterial genera…”

Line 283-284: The ratios were significantly higher in SBS and GLP2.SBS rats than those in Sham rats.”

Line 322: “Significant decrease of OTU counts and α diversity were observed in the colonic contents of SBS and GLP2.SBS rats which revealed…”

Line 352: “…alteration of the Firmicutes phylum in SBS and GLP2.SBS rats was the decrease of the…”

For ANOSIM, pairwise comparisons (i.e., Sham vs SBS, Sham vs GLP2.SBS, and SBS vs GLP2.SBS) are more useful to understand differences between the three groups. These could be easily added by the addition of pairwise comparison bars to the ANOSIM graphs (like the ones in Figure 3 for each taxa).

Line 28: delete the first word, “while” of the sentence “While limited data are available” to make grammatically correct.

Line 39: The bacterial taxa “Actinomycetes” is only mentioned in the graphical abstract (figure 11) and the abstract, but not mentioned anywhere else. Please change to Actinobacteria, or add Actinomycetes to table 2 or figure 3. If keeping as Actinomycetes, is this taxa different between GLP2.SBS and Sham? If so, then please change line 39-40 to “The relative abundance of Actinomycetes, Firmicutes and proinflammatory Proteobacteria increased significantly in SBS and GLP2.SBS group rats, while the relative abundance of Bacteroidetes, Verrucomicrobia and Tenericutes decreased remarkably.”

Line 363:”…anti-tumor effect, intestinal mucosal barrier protection, and alleviating colonic inflammation.”

Line 364-367: “Yu Xu and colleagues summarized that Akkermansia…” This sentence is unclear to me. Do you mean “…Akkermansia could protect the intestinal mucosal barrier and inhibit the translocation of intestinal flora, therefore reducing the level of LPS in circulation and thus inhibiting the inflammatory response.” ?

Line 408: “…reducing the relative abundance of Eurotiomycetes, Tremellomycetes and Dothideomycetes in SBS rats.” This part of the sentence is misleading because there was actually no significant difference in these taxa between SBS and GLP2.SBS groups. Please delete this part of the sentence.

Figure 1D does not appear to show median and IQR as is stated in the legend

**FEEDBACK FOR EDITOR**

Your recommendation

Revise

Is the study design appropriate to answer the research question (including the use of appropriate controls), and are the conclusions supported by the evidence presented?

Yes

Are the methods sufficiently described to allow the study to be repeated?

Yes

Is the use of statistics and treatment of uncertainties appropriate?

Yes

Is the presentation of the work clear?

No, it needs some language corrections before being published

Comments: It remains unclear when “SBS rats” are often discussed if this term refers to both SBS (saline) group and GLP2.SBS group rats, or only SBS saline group. This distinction is important because it will change the interpretation of the results. Additionally, there are a few grammatical errors that are addressed in the comments to the authors, Figure 1D does not show median and IQR as stated in the figure legend, and the bacterial taxa Actinomycetes is only ever mentioned in the abstract and graphical abstract and should be included elsewhere in the paper if it is to be discussed.

Are the images in this manuscript (including electrophoretic gels and blots) free from apparent manipulation?

Yes

Confidential Comments to the Editor
